# Tomentosin selectively targets microglial pyroptosis to overcome fluoxetine-resistant depression: a network-based therapeutic discovery

**DOI:** 10.1038/s41398-026-04092-5

**Published:** 2026-05-15

**Authors:** Jin-Seok Lee, Ji-Yun Kang, Won-Yung Lee, Ji-Yeon Gu, Tae-Wook Woo, Chang-Gue Son

**Affiliations:** 1 Institute of Bioscience & Integrative Medicine, Daejeon University, Daejeon, Republic of Korea; 2 Research Center for CFS/ME, Daejeon Hospital of Daejeon University, Daejeon, Republic of Korea; 3 School of Korean Medicine, Wonkwang University, Iksan, Republic of Korea

**Keywords:** Pharmacology, Molecular neuroscience, Depression

## Abstract

Treatment-resistant depression (TRD), a clinically challenging issue of major depressive disorder (MDD), affects up to one-third of patients and is associated with elevated suicide risk and limited treatment options. Cumulative evidence highlights pathological microglial state and inflammasome-derived pyroptosis as key contributors to TRD pathophysiology. This study aimed to validate tomentosin as a network-based identified antidepressants and anti-microglial candidate and to define its mechanisms in suppressing microglial pyroptosis. Through a network-based multiscale interactome screening of a large terpenoid library exceeding 170,000 compounds, we identified tomentosin, a brain-penetrant sesquiterpene lactone with favorable drug-likeness and network relevance. In mice unresponsive to fluoxetine (called as FRD, fluoxetine-resistant depression), tomentosin (20 mg/kg) significantly alleviated depressive behaviors and normalized reactive microglial states in the anterior cingulate cortex (ACC). These pharmacological effects were observed in systemic and intracerebral inflammation-induced depressive mice models. We found that tomentosin mechanistically targeted the suppression of microglial NOD-like receptor protein-3 (NLRP3)/caspase-1/gasdermin D (GSDMD) signaling pathway. This inflammasome-specific suppressive effect was confirmed by the absence of pharmacological effects in caspase-1 knockout (Casp1 KO) mice. Underlying mechanisms were further validated through molecular interaction analyses, comparative studies with inhibitors, and overexpression vector transfections. Our findings suggest that tomentosin is a novel agent that selectively modulates inflammasome-associated microglia in FRD, primarily by suppressing pyroptosis in the ACC.

## Introduction

Major depressive disorder (MDD) is a debilitating mental illness affecting 4.4% of the global population [1]. Its prevalence has steadily increased over the past few decades, making it a leading cause of disability worldwide [2] Although antidepressants are prescribed widely, about 50% of patients with MDD do not respond to first-line antidepressants, and one-third remain unresponsive even after adding a second antidepressant [3, 4]. Treatment-resistant depression (TRD) has long been recognized as a critical challenge, with patients exhibiting a 3-fold higher risk of suicide and self-harm compared to those with non-resistant forms of depression [5].

The pathophysiology of TRD is believed to be multifactorial, involving impaired transmission of monoamine neurotransmitters, dysregulation of hypothalamus–pituitary–adrenal (HPA), neuroinflammation, and neuronal dysconnectivity [6, 7]. Then, increasing evidence supports the microglial-targeted interventions against TRD [8, 9]. Individuals with TRD have been shown to exhibit greater translocator protein (TSPO, a marker of microglial activity) density than healthy and non-TRD patients, particularly in the anterior cingulate cortex (ACC) and prefrontal cortex (PFC) [10–12]. One hundred sixty-three young individuals who attempted suicide showed smaller frontal regions compared to 323 who had not [13]. Activated microglia cause neuroinflammation and can induce programmed neuronal cell death, known as pyroptosis, around microglia in which gasdermin D (GSDMD) pores have formed [14]. Whole-blood mRNA profiling of TRD patients has revealed an upregulation of inflammasome-associated genes [15]. Unfortunately, the anti-inflammatory candidates for TRD have yielded largely disappointing results in RCTs, particularly with lack of microglia-specificity [16, 17].

On the other hand, network pharmacology-based approaches, particularly multiscale interactome analysis, have emerged as powerful tools to identify high-specific candidate compounds that act on disease-relevant pathways [18]. The plant-derived volatile terpenoids have recently drawn attention for their potential central nervous system (CNS) activity [19, 20]. Leveraging this approach, we explored associations between natural terpenoids and microglia-dominant TRD networks and identified tomentosin–a sesquiterpene lactone derived from Inulae Flos (*Inula japonica* Thunb.)–as a promising therapeutic candidate. Inulae Flos has been traditionally used for its antidepressant properties [21], and tomentosin has previously demonstrated anti-inflammatory, anti-neuroexcitotoxicity, and neuroprotective effects [22–24]. Moreover, recent evidence confirms that Tomentosin can cross the blood-brain barrier (BBB) [25], further supporting is potential as a central nervous system (CNS)-active compound.

Building upon our network-guided findings, we experimentally validated the therapeutic potential of tomentosin in a fluoxetine-resistant depression (FRD) model by focusing on microglia-specific mechanisms. To elucidate its mode of action, we employed an integrated approach combined in silico network pharmacology with serial in vivo models and in vitro functional assays using BV2 and HMC3 microglial cells. These findings establish a mechanistic foundation for the further development of tomentosin as a novel treatment candidate for TRD, *at least* fluoxetine-unresponsive depression.

## Material and methods

### Compound-target-disease network construction

**Fig. 1:**
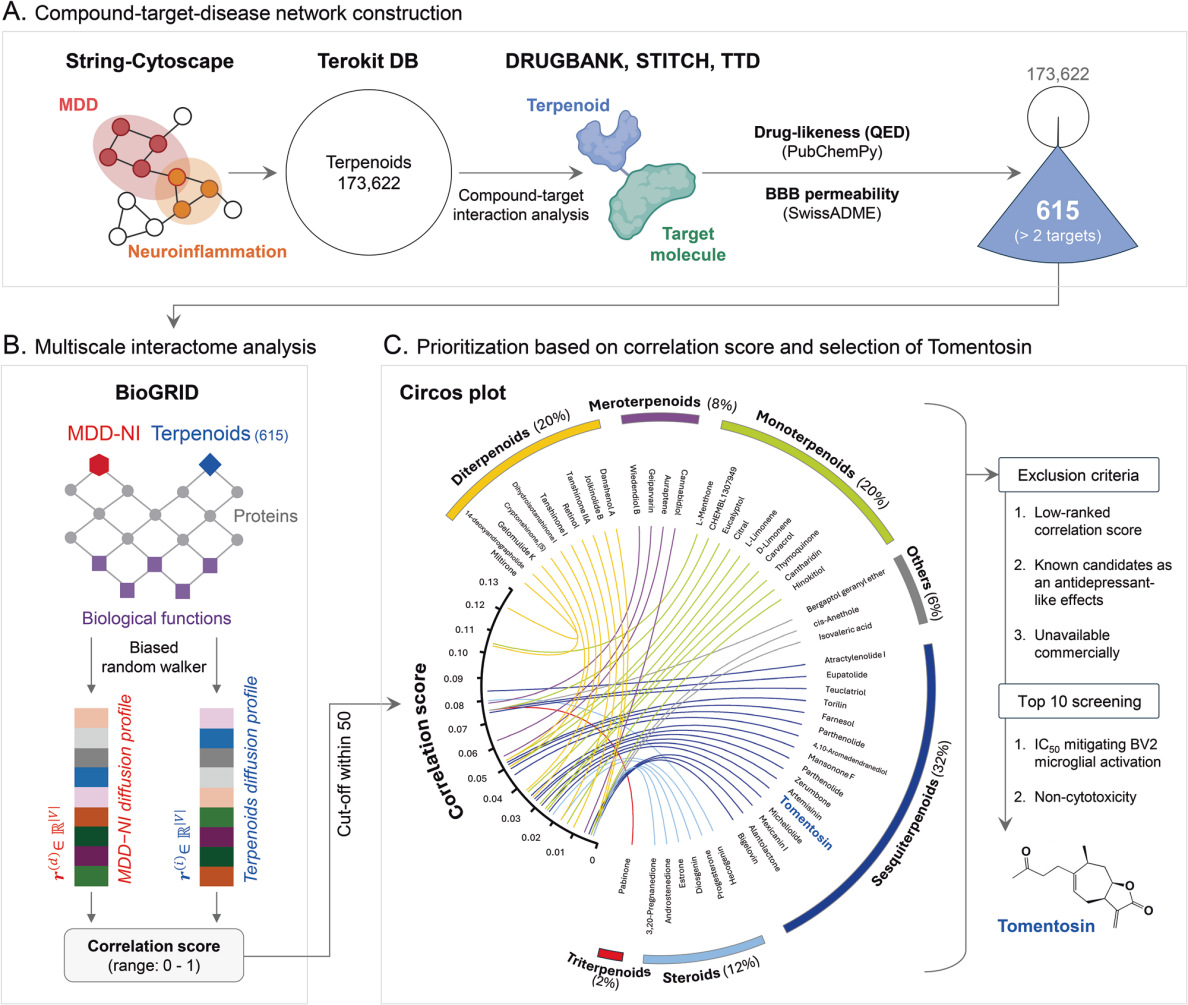
Network pharmacology-based identification of tomentosin as a novel candidate for treating TRD. Compound–target–disease network construction was performed with assembling MDD–associated neuroinflammatory proteins (left), and 173,622 terpenoids were mapped to validated targets and filtered by quantitative estimate of drug-likeness (QED) and blood–brain barrier (BBB) permeability, yielding 615 candidates (right) **A**. Disease (red) and terpenoid (blue) targets were embedded in a multiscale interactome analysis; diffusion profiles were computed and Pearson correlations (0-1) used to quantify network-level similarity **B**. The top 50 candidates were prioritized by correlation score and displayed with circos plot, and then tomentosin was selected by exclusion criteria and further in vitro assay-based experimental validation **C**.

To systematically identify potential terpenoid candidates targeting MDD-associated neuroinflammation, we first retrieved proteins associated with both “MDD” and “neuroinflammation” using PubMed-based text mining via the Cytoscape-StringApp. Terpenoid compounds were sourced from TeroKit (http://terokit.qmclab.com/), a specialized database for terpenome research [26]. Experimentally validated compound–target interactions were compiled from DrugBank, search tool for interactions of chemicals (STITCH), and Therapeutic Target Database (TTD). After mapping these compounds to valid PubChem IDs, the dataset was curated to include only those terpenoids for which sufficient target (≥2) information was available. BBB permeability was predicted using SwissADME (http://www.swissadme.ch/), and quantitative estimation of drug-likeness (QED) was computed via the PubChemPy module in Python. The QED threshold was set to 0.35 based on the comparative analysis [27], where this cutoff exhibited performance superior to Veber’s rule (Fig. 1A).

### Multiscale interactome analysis

A multiscale interactome was adopted [28], comprising protein–protein, protein–biological function, and biological function–biological function associations. Relevant interaction data were gathered from the Biological General Repository for Interaction Datasets (BioGRID) and the Database of Interacting Proteins. Diffusion profiles were then computed for terpenoid targets and for the MDD–neuroinflammation protein set by employing a matrix-based power iteration (biased random-walker) algorithm, which incorporated restart probabilities and scalar weights for node transitions. Pearson’s correlation was utilized to measure the similarity between each compound’s diffusion profile and the disease diffusion profile. Finally, the top k proteins or biological functions (with k = 20) most influenced in these diffusion profiles were extracted to construct a subnetwork representing key mechanisms. This threshold was determined based on a multiscale interactome study demonstrating that high-ranking nodes in diffusion profiles correspond to the most strongly propagated drug effects and account for the majority of the overall visitation frequency.

### Animals

A total of eighty male, specific pathogen-free C57BL/6J mice (8 weeks old, weighing 22–24 g) were purchased from Dae Han Bio Link (Co., Ltd., Eumseong, Korea). Additionally, 12 wild-type and 12 caspase-1 knockout (B6N.129S2-Casp1^tm1Fkv^/J) mice were obtained from The Jackson Laboratory (Bar Harbor, ME, USA). The mice were housed under controlled conditions at 23 ± 2 °C (temperature) and 55 ± 10% (humidity) using a thermohygrostat (ALFFIZ, BuSung Co., Ltd, Seoul, Korea), with a 12-h light-dark cycle (09:00 to 21:00) and *ad libitum* access to food (Cargill Agri Furina, Gyeonggido, Korea).

All animal care and experimental protocols were approved by the Institutional Animal Care and Use Committee of Daejeon University (DJUARB2024-019) and were conducted in accordance with the Guide for the Care and Use of Laboratory Animals, as published by the National Institutes of Health (NIH).

### Four mouse models and tomentosin administration

After a one-week acclimation period, the mice were allocated to three for the following experimental questions: (1) whether tomentosin overcomes FRD phenotype in an unpredictable chronic mild stress (UCMS) model (*n* = 24), (2) whether tomentosin exerts anti-depressive effects on lipopolysaccharide (LPS) injection-derived systemic inflammation model (*n* = 32), (3) whether the effects of tomentosin are dependent on the inflammasome using a Casp1 KO model challenged with LPS (*n* = 24). and (4) whether tomentosin inhibits microglial inflammasome-specific depression in a recombinant interleukin (rIL)-1β intracerebroventricular injection model (*n* = 24), respectively.

According to manufacturer’s guideline, the tomentosin was dissolved in 1% of DMSO. To prevent bias arising from different conditions, solvent and administrating route were evenly applied to all the designated groups.

### UCMS model

**Fig. 2:**
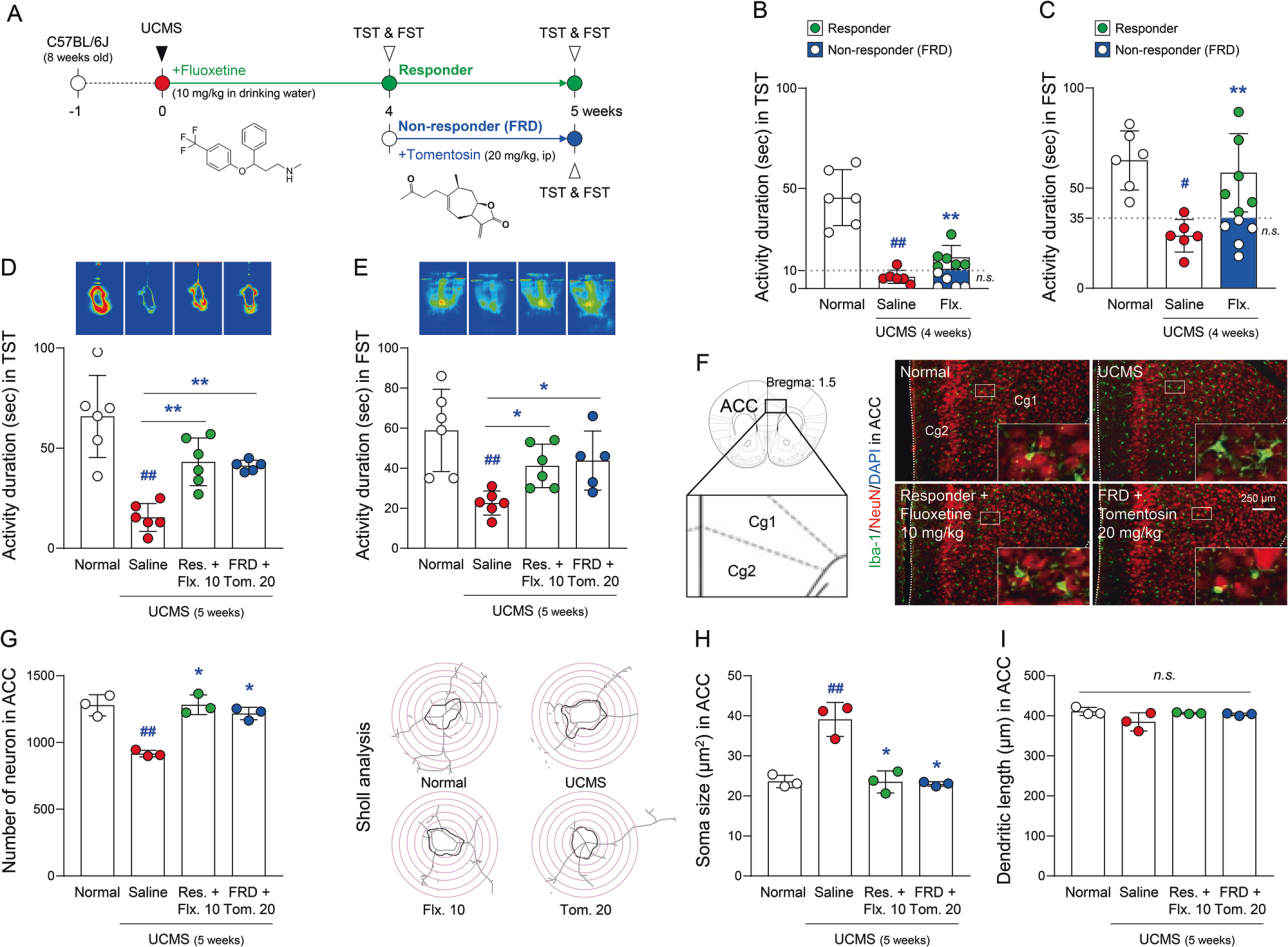
Effects of tomentosin on microgliopathy-dominant FRD in a UCMS mouse model. The schematic of the experimental schedule was designed **A**. After 4-week UCMS induction, the activity durations in TST **B** and FST **C** were evaluated to divide responder to fluoxetine and non-responder (FRD). After a 1-week UCMS with fluoxetine or tomentosin, the activity durations in TST **D** and FST **E** were re-evaluated. Iba-1 positive microglial activity and NeuN positive neuronal activity in the ACC were assessed by double-staining analysis **F**. The number of NeuN-positive cells in the ACC was quantified using ImageJ **G**. Microglial soma size **H** and process length **I** in the ACC were evaluated via Sholl analysis. Data are expressed as the mean ± SD (*n* = 3, 5 or 6/group). ^#^*p* < 0.05 and ^##^*p* < 0.01 com*p*ared to the normal mice; ^*^p < 0.05 and ^**^p < 0^.^01 compared to the UCMS-subjected mice.

Mice were randomly assigned to three groups as follows: normal (*n* = 6), UCMS (*n* = 6), and UCMS with fluoxetine (10 mg/kg, prepared in drinking water; PHR1394, Sigma-Aldrich, MO, USA; *n* = 12, one mouse death by drowning). After UCMS for 4 weeks, the fluoxetine-treated mice were divided into responder and non-responder called as TRD group following two behavioral tests such as tail suspension test (TST) and forced swimming test (FST). The TRD group was intraperitoneally administrated with tomentosin (20 mg/kg; BD013978, BLDpharm, Kaiserslautern, Germany) for one week, and then all of mice were re-examined behavioral tests evaluating depressive symptoms (Fig. 2A). The UCMS schedules were followed as shown in Table S1.

### Systemic LPS injection model

**Fig. 3:**
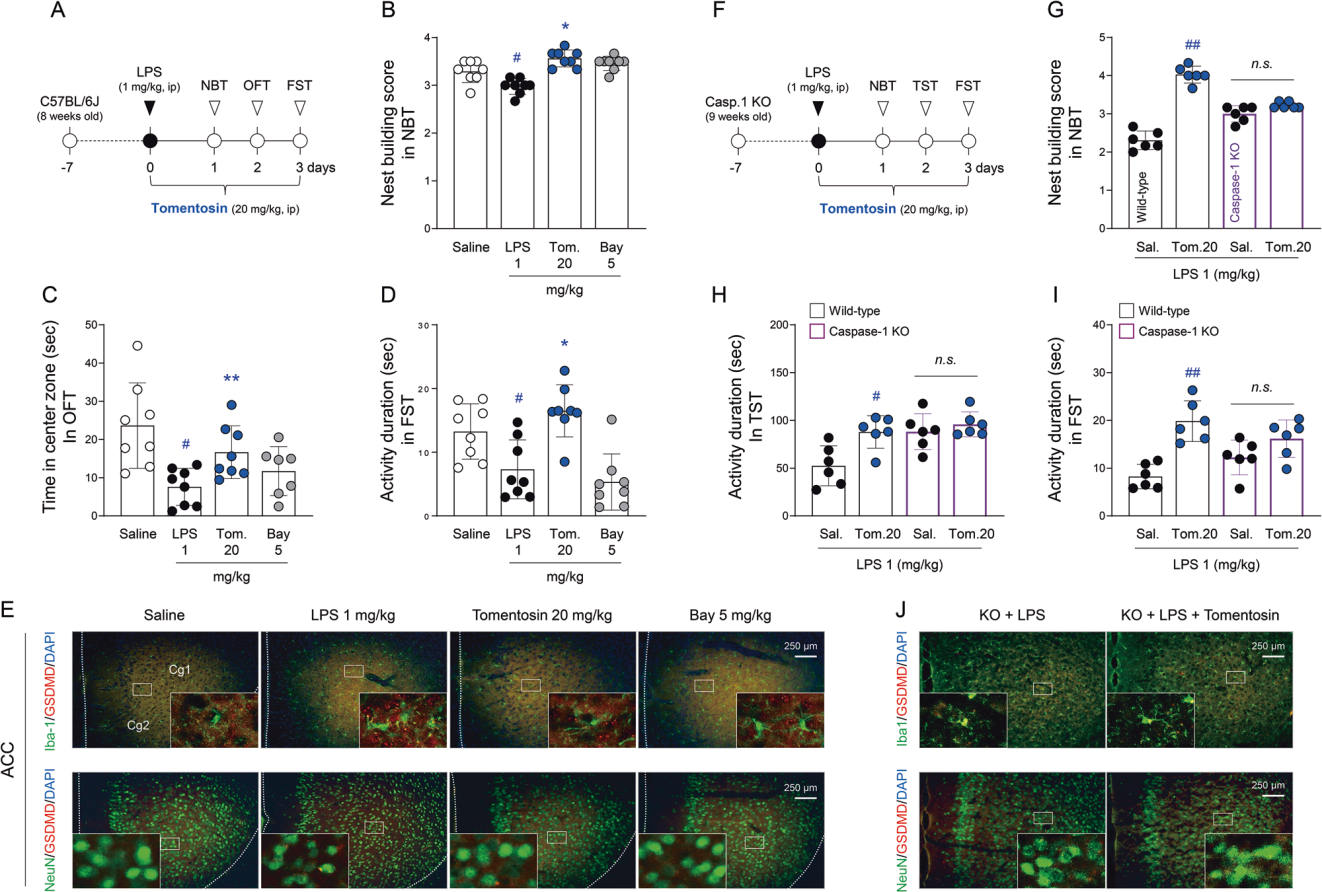
Effects of tomentosin on inflammation-mediated depression in systemic LPS-injected and Casp1 KO mouse models. The schematic of the experimental schedule in the LPS-injected mouse model was designed **A**. After intraperitoneal LPS injection, the nest-building score in NBT **B**, time spent in the center zone in OFT **C**, and active duration in FST **D** were evaluated in sequence. In the ACC region, microglial inflammasome was assessed by Iba-1/GSDMD and neuronal pyroptosis was examined by NeuN/GSDMD double-positive signals **E**. The experimental design to validate the anti-inflammasome effects of tomentosin was established using a Casp1 KO model **F**. The nest-building score in the NBT **G**, time spent in the center zone in the OFT (**H**), and active duration in the FST **I** were evaluated. In the ACC, microglial inflammasome and neuronal pyroptosis were examined using Iba-1/GSDMD and NeuN/GSDMD double-staining **J**. Data are expressed as the mean ± SD (*n* = 3, 6 or 8/group). ^#^*p* < 0.05 and ^##^*p* < 0.01 com*p*ared to the saline-injected mice; ^*^p < 0.05 and ^**^p < 0^.^01 compared to the LPS-injected mice.

Mice were randomly assigned to four groups (*n* = 8/group): saline, LPS (1 mg/kg, dissolved in normal saline; E. Coli O111:B4, L2630, Sigma-Aldrich, MO, USA), LPS with tomentosin, and LPS with BAY11-7082 (5 mg/kg, dissolved in normal saline; HY-13453, MCE, NJ, USA), respectively. After an injection of LPS, mice were intraperitoneally administrated with tomentosin or BAY11-7082 once daily for three days along with three sequential behavior tests such as nest building test (NBT), open field test (OFT) and FST (Fig. 3A).

### Casp1 KO mouse model under systemic LPS injection

To verify the regulatory effects of tomentosin on the NLRP3/caspase-1/GSDMD inflammasome pathway, wild-type and Casp1 KO mice were each divided into two treatment groups (*n* = 6 per group): LPS (1 mg/kg) with saline or LPS with tomentosin (20 mg/kg), resulting in a total of four experimental groups. Under the same conditions described above, the mice were subjected to three sequential behavioral tests: NBT, TST, and FST (Fig. 3F).

### Intracranial rIL-1β injection model

**Fig. 4:**
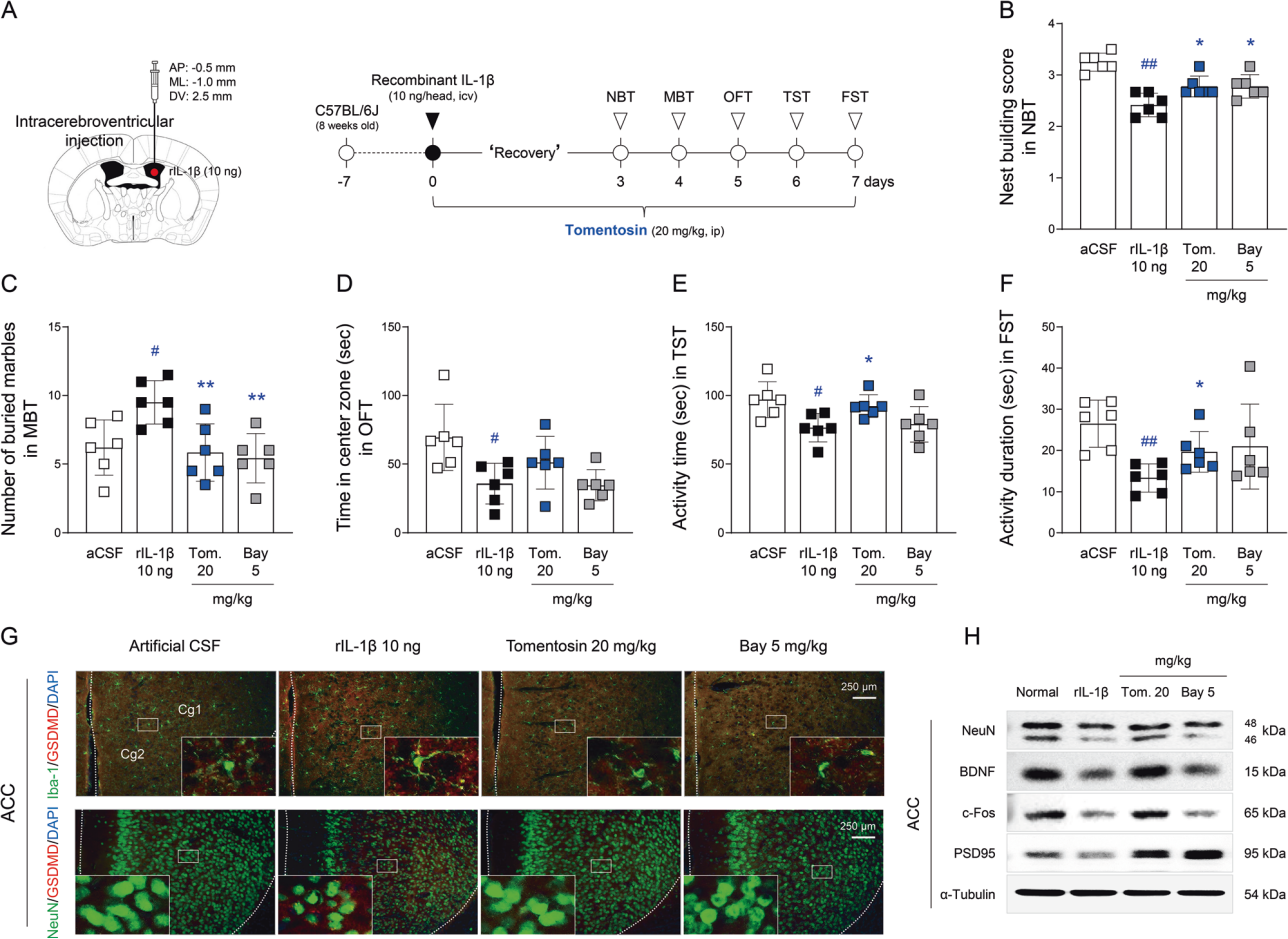
Effects of tomentosin on neuroinflammation-specific depression in the intracranial rIL-1β-injected mouse model. The schematic of the experimental schedule was designed **A**. After the intracranial rIL-1β injection, the nest-building score in NBT **B**, number of buried marbles in MBT **C**, time spent in the center zone in OFT **D**, active duration in TST **E** and FST **F** were evaluated in sequence. In the ACC region, microglial inflammasome was assessed by Iba-1/GSDMD and neuronal pyroptosis was examined by NeuN/GSDMD double-positive signals **G**. The protein levels of neuronal activity markers (NeuN, c-Fos, BDNF, and PSD95) in the ACC were examined by western blotting analysis **H**. Data are expressed as the mean ± SD (*n* = 3 or 6/group). ^#^*p* < 0.05 and ^##^*p* < 0.01 com*p*ared to the aCSF-injected mice; ^*^p < 0.05 and ^**^p < 0^.^01 compared to the rIL-1^β-^injected mice.

Mice were randomly assigned to four groups (*n* = 6/group): artificial cerebrospinal fluid (aCSF), rIL-1β (10 ng/head, dissolved in aCSF; 1857-LC, R&D Systems, MN, USA), rIL-1β with tomentosin, and rIL-1β with BAY11-7082. The rIL-1β was injected into the left lateral ventricle using a robotic stereotactic drill and injection system (Neurostar, Tuebingen, Germany) at a flow rate of 1 μL/min for 4 min. The injection coordinates relative to bregma were as follows: AP: −0.5 mm, ML: −1.0 mm, DV: 2.5 mm. To prevent backflow, the syringe was left in place for 5 min after the injection. After intracranial rIL-1β injection, mice were intraperitoneally administrated with tomentosin or BAY11-7082 once daily for seven days. After a 3-day recovery period following intracranial injection, five consecutive behavioral tests were performed in order of increasing stress intensity (NBT, MBT; marble burying test, OFT, TST, and FST), with a 24-h recovery interval between tests to minimize cumulative interference (Fig. 4A).

### Five behavioral tests and data analyses

All behavioral tests, including TST, FST, NBT, OFT, and MBT were followed as displayed in Figs. 2A–4A, and tracking data were recorded and calculated using a video camera connected to corresponding software (Smart 3.0, Panlab SL, Barcelona, Spain). All the performances were evaluated by researchers blinded to the experimental conditions. Detailed methods of all behavioral tests were described in supplementary materials.

### Immunofluorescence analysis in ACC

Microglia activity (Iba-1; ionized calcium-binding adapter molecule 1, IL-1β; interleukin 1 beta, and GSDMD), astrocytic activity (GFAP; glial fibrillary acidic protein and S100β; S100 calcium-binding protein β) and neuronal integrity (NeuN; neuronal nuclei) in ACC were assessed using immunofluorescent staining analysis. To prepare brain sections for analyzing histologically, three independent mice per group were transcranially perfused with 0.05% heparin (10 units/mL in PBS) on the final day of tomentosin injection, followed by 4% paraformaldehyde (pH 6.9). The removed brains were gradually cryoprotected in 10, 20, and 30% sucrose for 24 h each and were subsequently embedded in an optimal cutting temperature (OCT) compound (Leica Microsystems, Bensheim, Germany) in liquid nitrogen. They were cut into frozen coronal sections (30 μm) using a cryostat (CM3050_S, Leica), and sections were stored in free-floating buffer.

In brief, the brain sections were incubated with blocking buffer (5% normal chicken serum in PBS and 0.3% Triton X-100 for 1 h at 4 °C), and then were adapted with anti-rabbit Iba-1 (1: 400, 019-19741, Wako Biologicals), anti-rabbit IL-1β (1:100, ab9722, Abcam), anti-rabbit GFAP (1:200, Z0334, Dako), anti-rabbit GSDMD (1:100, #46451, Cell Signaling), anti-mouse NeuN (1:200, MAB377, Merck-Millipore), and anti-rabbit S100β (1:100, ab52642, Abcam) primary antibodies overnight at 4 C. After washing with ice-cold PBS, the sections were incubated with a goat anti-rabbit (1:400; Alexa Fluor 488, ab150077), goat anti-rabbit (1:400; Alexa Fluor 594, ab150080) and goat anti-mouse (1:400; Alexa Fluor 594, ab150116) secondary antibodies for 2 h at 4 °C. The sections were subsequently exposed to DAPI to stain the cell nuclei. Immunofluorescence imaging was performed on a Axio-phot microscope (Carl Zeiss, Jena, Germany) and Olympus IX71 microscope equipped with TH4-200 illumination and DP74 digital camera (Olympus, Tokyo, Japan). Fluorescent signals and morphological characteristics of stained cells (cell body size/cell, dendritic process/cell, or cell number/mm^2^) were analyzed using ImageJ/FIJI 1.54 (Sholl analysis) and Image-Pro Plus 6.0 software (NIH, Bethesda, MD, USA and Media Cybernetics, Inc. Rockville, USA).

### Blood-brain barrier permeability assay

To evaluate BBB penetrability of tomentosin, we used parallel artificial membrane permeability assay (PMBBB-096, BioAssay Systems, Eching, Germany), according to the manufacturer’s recommendations. Briefly, 500 μM of tomentosin and permeability controls (high and low) were dispensed into a donor plate well impregnated with BBB lipid solution. This donor plate was placed on the acceptor plate and then was incubated for 18 h at 37 °C. To minimize own absorbance of tomentosin and permeability controls, their equilibrium standards at 200 μM were prepared separately. The absorbance of acceptor solutions and equilibrium standards were measured using a NanoDrop device (Thermo Fisher Scientific, Madison, WI, USA). Permeability rate (cm/s) was calculated by equation provided and cut-off value of BBB permeability is 4.0 × 10^−6 ^cm/s.

### BV2 and HMC3 microglial cell experiments and tomentosin cytotoxicity

**Fig. 5:**
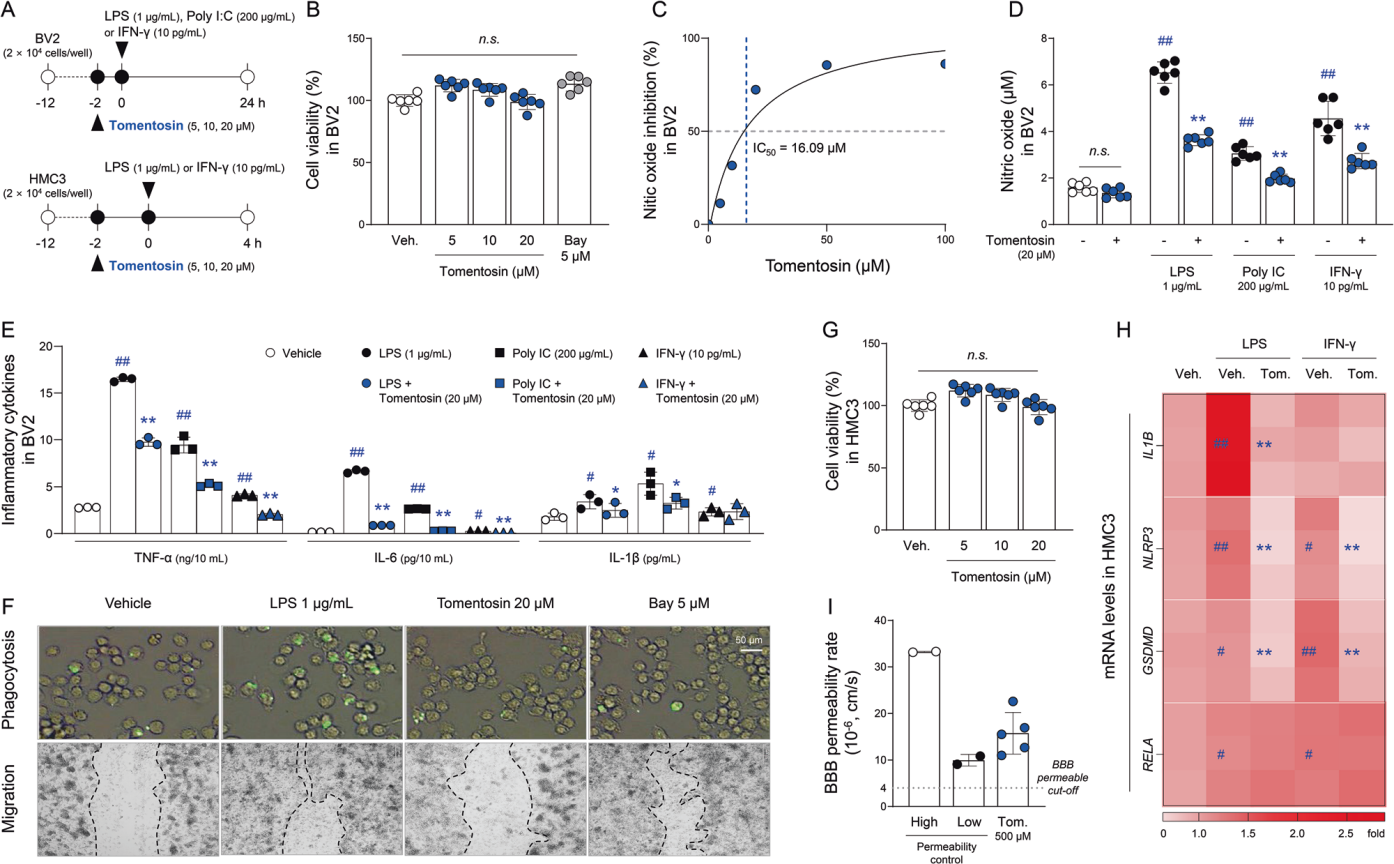
Effects of tomentosin on activated BV2 and HMC3 microglia and its byproducts. The schematic of the experimental schedule was designed **A**. The cytotoxicity of tomentosin and bay were measured by the WST-8 cell viability assay in the BV2 **B**. IC_50_ value of the tomentosin against LPS-induced NO productions was calculated **C**. Levels of NO, TNF-α, IL-6, and IL-1β under various stimulations (LPS, poly I:C, and IFN-γ) were measured **D** and **E**. Under LPS exposure, phagocytic activity was assessed using a FITC-fluorescent phagocytosis assay, and migratory activity was evaluated after 24 h, followed by semi-quantification in BV2 cells **F**. The cytotoxicity of tomentosin in HMC3 cells was further assessed by the WST-8 assay **G**. In HMC3 human microglial cells stimulated with two inducers (LPS or IFN- γ), the mRNA expression levels of inflammatory genes (*IL1B, NLRP3, GSDMD*, and *RELA*) were evaluated by RT-PCR **H**. Finally, the BBB permeability rate of tomentosin was assessed **I**. Data are expressed as the mean ± SD (*n* = 3 or 6/group). ^#^*p* < 0.05 and ^##^*p* < 0.01 com*p*ared to the vehicle-treated cells; ^*^p < 0.05 and ^**^p < 0^.^01 compared to the stimulator-exposed cells.

To further investigate inhibitory effects of tomentosin on microglia specifically, the mouse BV2 (ATCC, CRL-2468, VA, USA) and human HMC3 (ATCC, CRL-3304, VA, USA) microglial cell line was utilized (Fig. 5A). The cells were maintained at 37 °C under 5% CO_2_ and cultured in media (DMEM, LM001-05; WelGENE Inc., Daegu, Korea) supplemented with 10% FBS (S001-01, WelGENE Inc.) and 1% antibiotic-antimycotic solution (LS203-01, WelGENE Inc.).

To confirm whether tomentosin has microglial cytotoxicity, the BV2 and HMC3 cells (2 × 10^4^ cells/well) were seeded into a 96-well microplate for 12 h, and then tomentosin was treated for 26 h or 6 h, respectively. The cytotoxicity was evaluated using a WST-8 assay kit (EZ-Cytox, DoGenBio, Seoul, Korea). The absorbance was measured at 450 nm using a UV spectrophotometer (Molecular Devices, Sunnyvale, CA, USA).

### Microglial inflammatory- and inflammasome-associated assay

To obtain half-maximal inhibitory concentration (IC_50_) value of tomentosin against microglial nitric oxide (NO) production, the BV2 cells (2 × 10^4^ cells/well) were treated with tomentosin (5 to 100 μM) for 2 h before exposure to LPS (1 µg/mL, L2630, Sigma-Aldrich, MO, USA), poly I:C (200 μg/mL, P1530, Merck-Millipore, NJ, USA) or IFN-γ (10 pg/mL, 51-27606E, BD Biosciences, CA, USA). After incubation for 24 h, the supernatants were mixed with an equal volume of Griess reagent (1% sulfanilamide/0.1% N-(1-naphthyl)-ethylenediamine dihydrochloride/2.5% H_3_PO_4_) to determine levels of NO. After incubation for 15 min at 37 °C, the absorbance was measured at 540 nm using a UV spectrophotometer (Molecular Devices).

The BV2 and HMC3 cells were seeded into 6-well plates (2 × 10^4^ cells/well) and then incubated for 12 h. The cells were treated with tomentosin (20 μM) for 2 h before exposure to LPS (1 µg/mL) for 24 h. In BV2 experiment, cytokine levels of tumor necrosis factor-α (TNF-α, DY140), IL-6 (DY406), and IL-1β (DY400) in the supernatant were determined using an ELISA duo-set kit (R&D Systems, MN, USA). The absorbance was read at 450 nm using a UV spectrophotometer (Molecular Devices). In the HMC3 experiments, the mRNA levels of *IL1B*, *NLRP3*, *GSDMD*, *RELA*, and *GAPDH* were analyzed using real-time quantitative PCR (RT-qPCR). Total RNA was extracted using a RNeasy Mini Kit (Qiagen, CA, USA), and cDNA was subsequently synthesized using a High-Capacity cDNA Reverse Transcription Kit (Thermo Fisher Scientific, MA, USA). Quantitative PCR was performed using SYBR Green PCR Master Mix (Thermo Fisher Scientific, MA, USA), and PCR amplification was conducted according to a standard protocol on a Rotor-Gene Q real-time PCR system (Qiagen, CA, USA). The detailed primer sequences are provided in Table S3.

### Microglial phagocytosis and migration assay

To confirm inhibitory activity of tomentosin against pathological microglial phenotypes, we assessed phagocytic activity using a phagocytosis assay kit (500290, Cayman Chemical, MI, USA). Under the same conditions as for the cytokine assay, the BV2 cells were exposed to latex beads (1:200) conjugated with rabbit IgG-fluorescein 5-isothiocyanate (FITC) for 30 min. After fixation with 4% PFA, the phagocytosed fluorescent beads were counted using a microscope.

For assessing migration ability, the cells were wounded using a sterile 20 μL pipette tip. After incubation for 24 h, the migratory activity was determined by measuring the relative changes in the width of the wounds using a microscope. The degree of cell migration is expressed as the percentage of that of vehicle-treated cells.

### Western blot analysis of microglial cell lysates and ACC homogenates

Protein expression of BV2 microglial cell lysates for the inflammatory (TLR4; toll-like receptor 4, iNOS; inducible nitric oxide synthase, NF-κB; nuclear factor kappa, ERK; extracellular signal-regulated kinases, and STAT3; signal transducer and activator of transcription 3) and inflammasome (NLRP3; NLR family pyrin domain containing 3, ASC; apoptosis-associated speck-like protein, caspase-1, GSDMD, and IL-1β) signaling pathway were assessed. Microglial activity (Iba-1), neuronal activity (NeuN, c-Fos, BDNF; brain-derived neurotrophic factor, and PSD95; postsynaptic density protein 95), and programmed cell death (pro- and cleaved caspase 1 or 3) in mouse ACC tissue was evaluated by western blotting analysis.

In brief, the harvested cells were lysed using a protein extraction solution (Pro-Prep, iNtRON Biotechnology, Sungnam, Korea) and removed ACC tissues were homogenized in radioimmunoprecipitation assay (RIPA) buffer (R0278, Sigma, MO, USA), supplemented with protease and phosphatase inhibitor cocktails (#1861284, Thermo Scientific, MA, USA). After equalizing the protein concentrations using a bicinchoninic acid protein assay kit (BCA1 and B9643, Sigma-Aldrich, MO, USA), the lysates and homogenates were separated by 10% polyacrylamide gel electrophoresis and then transferred to polyvinylidene fluoride (PVDF) membranes. To minimize non-specific binding, the membranes were blocked in 5% bovine serum albumin (BSA) for 1 h. The membranes were incubated overnight at 4 °C with primary antibodies, including antibodies against the anti-mouse NeuN (1:1000, MAB377, Merck-Millipore), anti-mouse c-Fos (1:1000, ab208942, Abcam), anti-rabbit BDNF (1:1000, ab108319, Abcam), anti-mouse PSD95 (1:1000, ab13552, Abcam), anti-rabbit NLRP3 (1:1000, ab214185, Abcam), anti-mouse apoptosis-associated speck-like protein (ASC, 1:1000, #67824, Cell Signaling), anti-rabbit caspase-1 (1:1000, NBP1-45433, Novus), anti-rabbit GSDMD (1:1000, #46451, Cell Signaling), anti-rabbit IL-1β (1:1000, ab9722, Abcam), anti-rabbit caspase-3 (1:1000, #9662, Cell Signaling), anti-rabbit cleaved caspase-3 (1:1000, #9664, Cell Signaling), anti-rabbit TLR4 (1:1000, #14358, Cell Signaling), anti-rabbit iNOS (1:1000, PA1-036, Thermo Fisher), anti-rabbit NF-κB (1:1000, #8242, Cell Signaling), anti-rabbit phospho-NF-κB (1:1000, ab86299, Abcam), anti-rabbit ERK (1:1000, #9102, Cell Signaling), anti-rabbit phospho-ERK (1:1000, #9101, Cell Signaling), anti-mouse STAT3 (1:1000, MA1-13042, Thermo Fisher). anti-mouse phospho-STAT3 (1:1000, MA5-15193, Thermo Fisher) and anti-mouse α-tubulin (1:2000, ab7291, Abcam). The membranes were incubated with an HRP-conjugated anti-rabbit or anti-mouse antibody (GeneTex, Inc., Irvine, CA) for 1 h. The bands were visualized with an advanced enhanced chemiluminescence (ECL) kit. The intensity was analyzed by ImageJ version 1.46 (NIH, Bethesda, MD, USA).

### Molecular docking analysis associated with inflammasome-related proteins

Molecular docking simulations were performed to identify potential targets and validate specific binding modes through a multi-stage approach. The 3D structures of target proteins and ligand compounds, including tomentosin (PubChem CID: 155173), were retrieved from the RCSB PDB (Table S2), AlphaFold Protein Structure Database, or PubChem database, respectively. Prior to analysis, all ligand structures were energy-minimized and converted to PDBQT format using OpenBabel. Blind docking was initially performed on multiple candidate proteins using both the CB-Dock2 server and local AutoDock Vina software.

Subsequently, site-specific docking was conducted to elucidate the specific inhibitory mechanism against GSDMD using AutoDock Vina with the AlphaFold-predicted GSDMD structure (Chain A). For this specific analysis, a grid box was explicitly defined around the Cys191 residue and the known inhibitory pocket with an exhaustive parameter of 64. The top-scoring binding poses were identified based on binding affinity, and molecular interactions were visualized using PyMOL 3.0.4 and LigPlus software.

### Overexpressed GSDMD vector construction and cell transfection

To verify the working mechanism of tomentosin, the clonal gene (pCMV-Puro-Gsdmd, insert length: 1464 bp) for GSDMD overexpression (NM_026960) was constructed using a Twist vector (Twist Bioscience, CA, USA).

The BV2 microglial cells were seeded at 2 × 10^4^ cells/well into the 6 well plates and then incubated until grown to 70 ~ 80% confluency. The cells were transfected with 1 μg of plasmid DNA using Lipofectamine 3000 (L3000015, Thermo Fisher Scientific, MA, USA) diluted in Opti-MEM reduced serum medium (31985-062, Gibco, MA, USA) for 24 h. Puromycin (2 μg/mL) was treated to select transfected cell for 24 h. Underwent verification whether transfected using Western blotting analysis, the GSDMD overexpressed BV2 microglial cells were seeded at 2 × 10^4^ cells/well into the 6 well plates, and after 12 h, the LPS (1 μg/mL) and/or tomentosin (20 μM) were treated for 24 h to evaluate the inhibitory activity against GSDMD protein expression.

### Comparative with LDC7559 inhibitor and chemical stability evaluation

To compare the inhibitory activity of tomentosin with LDC7559 (GSDMD inhibitor, HY-111674, MCE NJ, USA) against activated microglia, cells were treated with tomentosin (20 μM) or LDC7559 (5, 10 and 20 μM) for 2 h before exposure to LPS. In addition, to evaluate the chemical stability, the tomentosin was on heating at 40 and 45 °C or acidification with HCl at pH 3.0, and then cells were treated with heated or acidified tomentosin (20 μM) for 2 h before exposure to LPS. After exposing 24 h to LPS, cell supernatants were mixed with Griess reagent to measure NO levels, and absorbance was measured at 540 nm using a UV spectrophotometer. Under the same conditions without LPS stimulation, the cytotoxicity of above was evaluated using a WST-8 assay kit. The absorbance was measured at 450 nm using a UV spectrophotometer (Molecular Devices).

### Statistical analysis

All results are expressed as the mean ± standard deviation (SD). Statistical significance was analyzed by unpaired Student’s t-test or one-way analysis of variance (ANOVA) followed by post hoc analysis by *Dunnet* t-test using Prism 7 software (GraphPad). Differences at *p* < 0.05 were considered significant.

## Results

### Network pharmacology-based identification of tomentosin

To discover novel terpenoids with potential therapeutic effects against neuroinflammation-driven MDD, we performed a network pharmacology analysis. Out of 173,622 terpenoids surveyed, their interactions with target molecules were analyzed using predictive tools. In addition to evaluating their CNS-pharmacological applicability, we computed their drug-likeness (based on QED scores incorporating molecular weight, solubility, hydrogen bond of acceptors/donors, polar surface area, rotatable bonds, and aromatic ring) and BBB permeability. A total of 615 candidates were identified that met all three of the following criteria: (1) molecular target diversity (>2), (2) drug-likeness (>0.3), (3) BBB penetrability (“pass”) (Fig. 1A).

Next, we performed a multiscale interactome analysis, calculating correlation scores (0–1) by diffusion profiles for each terpenoid target and comparing them with MDD-associated neuroinflammatory proteins (Fig. 1B). Among these, the top 50 candidates were displayed in circos plot, with the largest proportion of sesquiterpenoids (32%) (Fig. 1C). Next, the candidates were prioritized by three exclusion criteria: (1) low correlation score, (2) known antidepressant effects, and (3) unavailable commercially. Then, the top 10 candidates were evaluated by in vitro screening that considered IC_50_ against NO production in LPS-stimulated microglia, and non-cytotoxicity. Finally, we identified that tomentosin has a favorable QED (0.438), strong network correlation (score = 0.03), and low IC_50_ (16.1 μM) value, making it a compelling candidate for further investigation.

### Tomentosin alleviates FRD pathophysiology

Mice exposed to UCMS for 4 weeks exhibited predominant MDD-like behaviors compared to normal group, as evidenced by significant reductions in activity duration of TST (*p* < 0.01) and FST (*p* < 0.05). Among these UCMS-subjected 11 mice, six mice showed positive responses to treatment of antidepressants (fluoxetine, 10 mg/kg) from their behavioral tests, meanwhile other 5 mice didn’t respond to fluoxetine treatment (referred to FRD group) (Figs. 2B, C and S2A, B). However, when FRD mice were administered with tomentosin (20 mg/kg) for a week, their depression-like behaviors were significantly alleviated, as level of fluoxetine-responding mice (*p* < 0.05 or *p* < 0.01; Fig. 2D, E).

Regarding pathological alterations, UCMS induced a significant increase in Iba-1-positive signals (a marker of microglia) (*p* < 0.01; Figs. 2F and S3A) and microglial soma size (*p* < 0.01; Figs. 2G to I and S3E, F), alongside an elevated number of GFAP/S100β-positive reactive astrocytes (*p* < 0.05; Fig. S4A, B). These changes were associated with a significant decline in neuronal integrity within the ACC, evidenced by a reduction in NeuN-positive neurons (*p* < 0.05; Figs. 2F and S3A) and the expression of neuroactive proteins, including BDNF, c-Fos, and PSD95 (*p* < 0.01; Fig. S1D and F). Furthermore, a marked elevation in serum C-reactive protein (CRP) levels (*p* < 0.01; Fig. S1C) was observed in the UCMS group. While both fluoxetine and tomentosin successfully reversed UCMS-induced neuronal dysfunctions (*p* < 0.05 or *p* < 0.01 for all alteration), only tomentosin exhibited a systemic anti-inflammatory effect by attenuating serum CRP levels (*p* < 0.01). Notably, tomentosin did not affect the reactive astrocytes.

### Tomentosin inhibits microgliopathy-related depressive behaviors

Given that microgliopathy is an emerging pathophysiological hypothesis of TRD, we adapted inflammation-mediated MDD mouse models. Mice exposed to systemic LPS (1 mg/kg) injections exhibited depressive- and anxiety-like behaviors compared to saline-injected controls, as evidenced by significant reductions in nest-building scores (NBT; *p* < 0.05), time spent in the central area (OFT; *p* < 0.05), and active duration (FST; *p* < 0.05). These behaviors were considerably improved by administration of tomentosin compared to LPS-injected mice (*p* < 0.05 or *p* < 0.01; Figs. 3B to D and S2C, D). Notably, while the antidepressant and anti-neuroinflammatory effects of tomentosin were robust in wild-type mice (*p* < 0.05 or *p* < 0.01), these effects were completely abolished in LPS-challenged Casp1 KO mice (Figs. 3G to I and S2E, F).

To further evaluate the inhibitory effects of tomentosin under brain-specific neuroinflammatory conditions, mice were subjected to intracranial injection of rIL-1β (10 ng/head). Tomentosin treatment significantly improved depressive behaviors in four tests (NBT, MBT, TST, and FST) compared to aCSF-injected mice, except OFT (*p* < 0.05 or *p* < 0.01; Figs. 4B to F and S2G to I). Overall, the effect size of tomentosin was generally superior to that of BAY11-7082 (5 mg/kg).

### Tomentosin suppresses microglial-derived neuronal pyroptosis in the ACC

We further examined the selective inhibitory activity of tomentosin on microglial-mediated inflammasome and neuronal pyroptosis in the ACC. Systemic LPS challenge significantly enhanced microglial inflammasome signaling, as evidenced by increased Iba-1/IL-1β co-localization (*p* < 0.01; Fig. S4C and D), Iba-1 protein expression (*p* < 0.05; Fig. S5A and B), and Iba-1/GSDMD double-positive signals (*p* < 0.01; Figs. 3E and S3B) compared to saline-injected mice. Sequentially, neuronal pyroptosis (NeuN-positive neurons around GSDMD, *p* < 0.01; Figs. 3E and S3B) and neuronal dysfunction (NeuN, BDNF, c-Fos, and PSD95 protein expressions, *p* < 0.05 or *p* < 0.01; Fig. S5A, B) were observed. While tomentosin significantly attenuated these pathological alterations (*p* <*p* < 0.05 or *p* < 0.01 for all signals), its protective effects were completely abolished in Casp1 KO mice (Figs. 3J and S3C, 4E).

Correspondingly, mice subjected to intracranial rIL-1β injection exhibited microglial GSDMD pore formation, as evidenced by increased Iba-1/IL-1β (*p* < 0.01; Fig. S4F and G) and Iba-1/GSDMD (*p* < 0.01; Figs. 4G and S3D) positive signaling in the ACC. In parallel, neuronal pyroptosis, characterized by NeuN/GSDMD double-positive signals (*p* < 0.01; Figs. 4G and S3D), and reduced expression of neuroactive proteins (*p* < 0.01; Figs. 4H and S5E) were observed in the ACC. Tomentosin blocked microglial-derived pyroptosis compared to aCSF-injected mice (*p* < 0.05 or *p* < 0.01 for all signals). Specifically, along with FRD model (Fig. S1D to F), tomentosin inhibited the cleavage of caspase-1 in the ACC of both MDD models (Fig. S5A to D). however, this was not observed for caspase-3.

### Tomentosin mitigates activated phenotype of microglia and their byproducts

To evaluate whether tomentosin effectively suppress stimulus-evoked microglial inflammatory responses, the mouse BV2 microglial cells were applied. As expected, the IC_50_ value of tomentosin for the LPS-induced NO levels was 16.09 μM in this cell line, denoting that effective and non-cytotoxic dose of tomentosin is around 20 μM (Fig. 5B and C). As a marker of activated microglia, NO production was notably increased by non-cytotoxic dose of three inducers (Fig. S6A), such as LPS (approximately 4.1-fold), poly I:C (approximately 2-fold), and IFN-γ (approximately 2.9-fold), whereas these were significantly inhibited by tomentosin treatment (*p* < 0.01 for each; Fig. 5D). These microglial inhibitory effects were concordantly supported by suppressing production of inflammatory cytokines (TNF-α, IL-6, and IL-1β; *p* < 0.05 or *p* < 0.01; Fig. 5E). Furthermore, tomentosin strongly reduced both FITC-bead phagocytosis and migration into scratched areas (*p* < 0.01 for both; Figs. 5F and S6B, C).

For cross-validation, the activity of tomentosin was further assessed in HMC3 human microglial cells. Non-cytotoxic doses of tomentosin (Fig. 5G) significantly suppressed the upregulation of inflammasome-related genes (*IL1B, NLRP3*, and *GSDMD*) induced by LPS or IFN-γ (*p* < 0.05 or *p* < 0.01; Figs. 5H and S7D), although no significant effect was observed for *RELA* (p65).

We confirmed that tomentosin had a high BBB permeability rate (approximately 4 times cut-off levels) which was compared to equal dose of permeability controls (Fig. 5I).

### Tomentosin normalizes molecular alterations of activated microglia

**Fig. 6:**
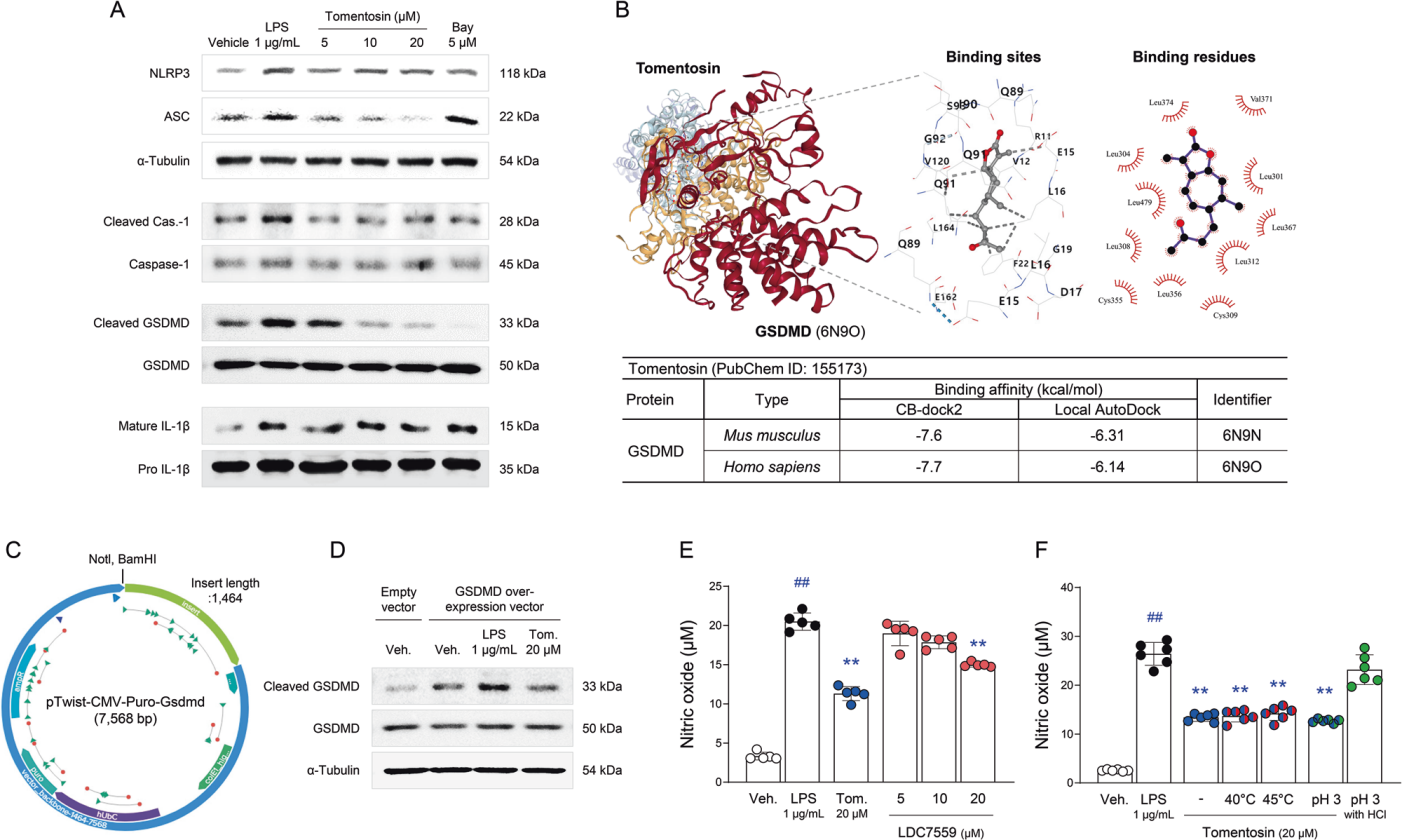
Effects of tomentosin on activated microglial-derived molecular alterations. Levels of protein-associated with NLRP3/caspase-1/GSDMD inflammasome pathway in LPS-stimulated BV2 microglia were examined by Western blotting analysis **A**. Binding affinity, binding sites, and amino acid residues of both mouse and human GSDMD to tomentosin were analyzed by Vina binding energy score **B**. The pTwist-CMV-Puro-Gsdmd vector map was displayed **C**. Under conditions of GSDMD overexpression and LPS stimulation, the inhibitory effect of tomentosin on GSDMD cleavages was assessed **D**. These inhibitory effects were compared to LDC7559 (a potent GSDMD inhibitor) **E**. Chemical and effective stability of tomentosin was assessed under various environmental conditions (e.g., heating up to 45 °C and acidic pH 3.0) **F**. Data are expressed as the mean ± SD (*n* = 5 or 6/group). ^##^*p* < 0.01 compared to the vehicle-treated cells; ^**^p < 0.01 com*p*ared to the LPS-treated cells.

To figure out the underlying mechanisms of tomentosin, we investigated microglial neuroinflammation-related pathways. LPS exposure led to sequential activation of molecular cascades involved in NF-κB translocation and the NLRP3 inflammasome, while they were substantially alleviated by tomentosin treatment (*p* < 0.05 or *p* < 0.01; Fig. 6A). Tomentosin primarily targeted the NLRP3/caspase-1/GSDMD pathway rather than the TLR4/NF-κB pathway, as indicated by the protein expression patterns (Fig. S7A to C).

From the Gene Ontology and KEGG pathway enrichment analyses of tomentosin’s network targets, we identified major pathways related to inflammation and cell survival/apoptosis (Fig. S1A and B).

### Tomentosin exerts high binding interaction with GSDMD and is competitive to inhibitor

To verify tomentosin’s interaction with GSDMD, molecular docking analysis was performed using AutoDock Vina, which assesses binding affinities with multiple target proteins. Among the proteins tested, tomentosin exhibited the highest binding affinity for GSDMD (*Mus musculus*: −7.6 kcal/mol and *Homo sapiens*: −7.7 kcal/mol) surpassing other potential targets such as NLRP3 (−7.0 kcal/mol), IL-1β (−6.2 kcal/mol), caspase-1 (−5.6 kcal/mol), and STAT3 (−6.6 kcal/mol). The docking model revealed that tomentosin forms stable hydrophobic contacts within a specific binding pocket of GSDMD, a finding consistently supported by independent local docking simulations (Fig. 6B and Table S2). Moreover, comparative docking analysis further demonstrated that tomentosin occupies the same binding pocket as disulfiram, specifically targeting the reactive Cys191 residue (Fig. S8).

We additionally validated the microglial GSDMD-specific inhibition of tomentosin in intensified conditions of GSDMD expression. As expected, tomentosin significantly inhibited GSDMD cleavage in LPS-stimulated microglial cells underwent transfection of GSDMD overexpression vector (*p* < 0.01; Figs. 6C, D and S6D). Notably, when compared to a known GSDMD inhibitor (LDC7559), tomentosin showed an even stronger inhibition of microglial NO production (Figs. 6E and S6E), indicating competitive efficacy.

Regarding chemical stability, tomentosin maintained its microglial inhibitory effects even after heating to 45 °C or exposure to acidic conditions (pH 3.0), further supporting its potential as a robust therapeutic candidate (*p* < 0.01 for all; Figs. 6F and S6F).

## Discussion

Current therapeutic strategies for managing MDD remain controversial, particularly given that up to half of patients do not respond to first-line antidepressants enhancing serotonergic transmission [29]. Consistent with this high non-response rate to selective serotonin reuptake inhibitors (SSRIs), our current UCMS-induced MDD model also exhibited a subset of mice unresponsive to fluoxetine, categorized as fluoxetine-resistant depression (FRD) (Fig. 2A to C). Despite the widespread prescription of SSRIs, the continuously increasing prevalence of MDD has led to growing skepticism toward the serotonin-targeted treatments [30, 31]. Furthermore, a prospective positron emission tomography (PET) study reported no significant difference in 5-HT transporter binding between patients with TRD and those with non-TRD, suggesting that serotonergic dysfunction may not be the primary mechanism underlying TRD [32].

Accumulating data have proposed that microglia-driven neuroinflammation are key contributors to the pathophysiology of TRD [33–37]. Although previous clinical trials using anti-inflammatory agents (e.g., minocycline, infliximab) demonstrated limited overall efficacy, modest benefits were predominantly observed in TRD patients with elevated serum c-reactive protein (CRP) levels (>3 or 5 mg/L) [16, 17]. This suggests that targeting brain-specific neuroinflammation, particularly microglial activation, may offer a more effective therapeutic strategy. In this study, high serum CRP levels and depressive behaviors were observed in FRD model, and it was dramatically normalized by tomentosin (Fig. S1C). Microglia are essential for maintaining neuronal homeostasis; however, they polarize into activated phenotypes in pathological conditions [38, 39]. This ‘microgliopathy’, commonly links to neurotoxicity leading to functional and/or structural impairment, especially in regions of ACC and PFC [40, 41]. As expected, tomentosin treatment (20 mg/kg) notably attenuated microglial morphological changes in soma size and neuronal shrinkage (evidenced by reduced cell numbers and neuroactive proteins) in the ACC of FRD mice (Figs. 2D to I and S1), but it had no effect on reactive astrocytes (Fig. S4A and B). These findings are particularly relevant as TRD patients exhibit more severe microglial activation and immunological dysregulation in the PFC-ACC region than non-TRD patients [10, 42, 43].

Both ACC and PFC have been shown to exhibit heightened sensitivity to peripheral inflammatory signals—as reflected by elevated serum CRP levels—owing to their relatively weak BBB and elevated expression of receptors for inflammatory cytokines, including IL-1 and TNF-α [44, 45]. We initially employed a systemic LPS-induced inflammation model (Fig. 3A), in which tomentosin reduced microglia-associated neuroinflammation in the ACC and improved depressive-like behaviors (Fig. 3B to E), independent of sickness behavior (Fig. S2A to I). A recent comprehensive review integrating neuroimaging features of TRD indicated the reduced fronto-cingulate connectivity [46]. Particularly, the prefrontal regions are known to be areas where microglia are easily activated by chronic stress and inflammatory conditions [47, 48]. Based on the caspase-1-specific inhibitory activity of tomentosin (Fig. S1D and F), we validated its pharmacological specificity for pyroptosis using a Casp1 KO mouse model (Figs. 3F to J and S3C) and intracranial injection of rIL-1β (Figs. 4B to H and S3D). The ACC plays a central role in emotion regulation and self-referential processing, and its functional-structural deficits are commonly observed in suicidal individuals [49–51]. Postmortem evidence indicates that high microglial priming and inflammasome activation in the ACC contribute to suicidality in depressed individuals [41, 52]. These may be relevant to explain the increased suicide risk in TRD [53, 54], although a cause-and-effect relationship between microglia and suicidality has not yet been established. Additionally, in vitro assays demonstrated that tomentosin remarkably suppressed highly activated microglia (BV-2 and HMC3 cells) as well as their inflammatory and inflammasome-related molecular alterations (Fig. 5B to H).

Building on the above therapeutic potential of tomentosin, we further explored its molecular mechanisms, focusing on inflammasome-related microglial dysfunction. Recently, microglial NLRP3 inflammasome and its downstream pyroptotic signaling have been found as key mediators in depression [55–57]. A clinical study found that TRD patients and responsive patients could be clearly separated by differential expression of NLRP3-related genes in peripheral blood transcriptomic analyses [58]. Notably, antidepressant efficacy has been observed only in microglia-specific, not astrocyte-specific, *Nlrp3* knockout mice [59]. Typically, microglial NLRP3 inflammasome activation induces caspase-1-dependent GSDMD cleavage, resulting in microglial pyroptosis and the release of pro-inflammatory cytokines that exacerbate neuronal damage [14, 60]. As expected, tomentosin treatment suppressed GSDMD cleavage and microglia-derived IL-1β in the ACC across all inflammasome-related animal models, as shown by immunofluorescence staining (Figs. 3E, J, 4G and S4C to G), and further supported by reduced levels of NLRP3/caspase-1/GSDMD signaling-related genes and proteins (Figs. 5H and 6A). Molecular docking analysis revealed tomentosin’s strong binding affinity to GSDMD primarily via hydrophobic interactions (Fig. 6B), which are likely to interfere with its pore-forming N-terminal domain [61, 62]. Notably, tomentosin occupies the same binding pocket as the specific GSDMD inhibitor disulfiram and exhibits similar binding affinity by interacting specifically with the reactive Cys191 residue within the GSDMD N-terminal domain (Fig. S8). These interactions were validated under GSDMD-overexpressing conditions, where tomentosin exhibited superior inhibitory efficacy compared to a known GSDMD inhibitor (Fig. 6C to E). Importantly, tomentosin also exhibited strong thermal and acidic stability, further enhancing its pharmacological potential (Fig. 6F). A comprehensive summary of the proposed mechanism is illustrated in Fig. S9.

To find a novel candidate compound overcoming TRD, we applied a computational network-based pharmacology system, thus we could identify tomentosin having the highest correlation score targeted microglial pathology (Fig. 1). We initially focused on terpenoids due to their higher BBB penetrability compared to flavonoids and alkaloids [19], along with their structurally rich hydrocarbon chains bearing abundant bioactive functional groups [63]. Many essential oils comprising terpenoids are known to improve depressive/anxious moods [64], and terpenoids are considered promising antidepressant candidates because of their strong inhibitory properties against microglial-derived neuroinflammation [65]. Of note, terpenoids preferentially inhibit pathological microglial phenotypes without affecting the physiological function of resting microglia [66]. D-limonene, a monoterpene, evidenced the anti-anxiety effects by a phase 2 clinical trial (NCT06378957) [67]. Despite these powerful properties of terpenoids, discovering an optimal drug candidate remains challenging due to its largest diversity to be 5 times more than that of polyphenols and alkaloids [68, 69].

Although numerous natural compounds have been proposed as antidepressant candidates, few have been specifically investigated for use in TRD [70]. In CNS drug development, along with effect size, brain bioavailability and safety (i.e., non-toxicity) are equally important. Esketamine was approved in 2019 for TRD [71]; however, its clinical utility is limited by severe side effects such as dissociation, which affects over 40% of patients [72]. While minocycline is known to inhibit the microglial NLRP3 inflammasome, it has been criticized for exerting non-specific glial-suppressive effects even under physiological conditions [73, 74]. Tomentosin is abundant in the leaves of *Inula viscosa* and *Inula japonica*, with a high isolated yield (0.64%) [22]. Previous studies have reported its neuroprotective effects on animal models of cerebral ischemia and neuro-excitotoxicity [23, 24]. We confirmed that tomentosin has high BBB penetrability and does not interfere with the physiological function of resting microglia (Fig. 5D and I). In this study, BAY 11-7082 was used as a positive control because it inhibits IκBα phosphorylation and suppresses NF-κB-dependent inflammasome priming, thereby serving as an upstream comparator. Tomentosin demonstrated superior overall efficacy compared with this positive control (Figs. 3 and 4).

Our results suggest that tomentosin exerts a selective effect on microglia-derived inflammasome, making it a promising candidate for TRD. Notably, our AI-driven approach combined with experimental validation demonstrated both time efficiency and predictive accuracy in addressing unmet clinical needs for TRD drug discovery. Although this is the first study to explore the antidepressant potential of tomentosin specifically in the context of FRD, several limitations should be acknowledged: (1) serotonergic modulation was not evaluated; (2) long-term safety and pharmacokinetic profiles remain to be determined; (3) potential interactions with conventional antidepressants have not been explored.

In conclusion, our study provides strong preclinical evidence that tomentosin is a promising candidate for FRD, primarily through selective inhibition of pathological microglia in the ACC via suppression of NLRP3/caspase-1/GSDMD signaling. These findings support further translational efforts, with tomentosin positioned as a first-in-class compound. Future studies should explore its serotonergic activity, long-term safety, and efficacy in diverse TRD models. Moreover, comprehensive validation will require time-resolved molecular analyses, dose–response characterization, larger animal sample sizes, anhedonia-focused behavioral assessments, and the inclusion of female subjects to improve translational applicability.

## Supplementary information

Supplementary Materials

## Data Availability

The data that support the findings of this study are available from the corresponding author upon reasonable request.

## References

[1] Friedrich MJ. Depression is the leading cause of disability around the world. JAMA. 2017;317:1517. 28418490 10.1001/jama.2017.3826

[2] Collaborators C-MD. Global prevalence and burden of depressive and anxiety disorders in 204 countries and territories in 2020 due to the COVID-19 pandemic. Lancet. 2021;398:1700–12. 34634250 10.1016/S0140-6736(21)02143-7 PMC8500697

[3] Rush AJ, Trivedi MH, Wisniewski SR, Nierenberg AA, Stewart JW, Warden D, et al. Acute and longer-term outcomes in depressed outpatients requiring one or several treatment steps: a STAR*D report. Am J Psychiatry. 2006;163:1905–17. 17074942 10.1176/ajp.2006.163.11.1905

[4] Lee JS, Lee SB, Kim DW, Shin N, Jeong SJ, Yang CH, et al. Social isolation-related depression accelerates ethanol intake via microglia-derived neuroinflammation. Sci Adv. 2021;7:eabj3400. 34739315 10.1126/sciadv.abj3400 PMC8570606

[5] Lundberg J, Cars T, Loov SA, Soderling J, Sundstrom J, Tiihonen J, et al. Association of treatment-resistant depression with patient outcomes and health care resource utilization in a population-wide study. JAMA Psychiatry. 2023;80:167–75. 36515938 10.1001/jamapsychiatry.2022.3860 PMC9856735

[6] Cui L, Li S, Wang S, Wu X, Liu Y, Yu W, et al. Major depressive disorder: hypothesis, mechanism, prevention and treatment. Signal Transduct Target Ther. 2024;9:30. 38331979 10.1038/s41392-024-01738-y PMC10853571

[7] Kajumba MM, Kakooza-Mwesige A, Nakasujja N, Koltai D, Canli T. Treatment-resistant depression: molecular mechanisms and management. Mol Biomed. 2024;5:43. 39414710 10.1186/s43556-024-00205-y PMC11485009

[8] Yirmiya R, Rimmerman N, Reshef R. Depression as a microglial disease. Trends Neurosci. 2015;38:637–58. 26442697 10.1016/j.tins.2015.08.001

[9] Deng SL, Chen JG, Wang F. Microglia: a central player in depression. Curr Med Sci. 2020;40:391–400. 32681244 10.1007/s11596-020-2193-1

[10] Richards EM, Zanotti-Fregonara P, Fujita M, Newman L, Farmer C, Ballard ED, et al. PET radioligand binding to translocator protein (TSPO) is increased in unmedicated depressed subjects. EJNMMI Res. 2018;8:57. 29971587 10.1186/s13550-018-0401-9 PMC6029989

[11] Setiawan E, Attwells S, Wilson AA, Mizrahi R, Rusjan PM, Miler L, et al. Association of translocator protein total distribution volume with duration of untreated major depressive disorder: a cross-sectional study. Lancet Psychiatry. 2018;5:339–47. 29496589 10.1016/S2215-0366(18)30048-8

[12] Yang KC, Chou YH. Molecular imaging findings for treatment resistant depression. Prog Brain Res. 2023;278:79–116. 37414495 10.1016/bs.pbr.2023.03.003

[13] van Velzen LS, Dauvermann MR, Colic L, Villa LM, Savage HS, Toenders YJ, et al. Structural brain alterations associated with suicidal thoughts and behaviors in young people: results from 21 international studies from the ENIGMA Suicidal Thoughts and Behaviours consortium. Mol Psychiatry. 2022;27:4550–60. 36071108 10.1038/s41380-022-01734-0 PMC9734039

[14] Devant P, Kagan JC. Molecular mechanisms of gasdermin D pore-forming activity. Nat Immunol. 2023;24:1064–75. 37277654 10.1038/s41590-023-01526-w PMC12379974

[15] Cattaneo A, Ferrari C, Turner L, Mariani N, Enache D, Hastings C, et al. Whole-blood expression of inflammasome-and glucocorticoid-related mRNAs correctly separates treatment-resistant depressed patients from drug-free and responsive patients in the BIODEP study. Transl Psychiatry. 2020;10:232. 32699209 10.1038/s41398-020-00874-7 PMC7376244

[16] Nettis MA, Lombardo G, Hastings C, Zajkowska Z, Mariani N, Nikkheslat N, et al. Augmentation therapy with minocycline in treatment-resistant depression patients with low-grade peripheral inflammation: results from a double-blind randomised clinical trial. Neuropsychopharmacology. 2021;46:939–48. 33504955 10.1038/s41386-020-00948-6 PMC8096832

[17] Raison CL, Rutherford RE, Woolwine BJ, Shuo C, Schettler P, Drake DF, et al. A randomized controlled trial of the tumor necrosis factor antagonist infliximab for treatment-resistant depression: the role of baseline inflammatory biomarkers. JAMA Psychiatry. 2013;70:31–41. 22945416 10.1001/2013.jamapsychiatry.4 PMC4015348

[18] Lee WY, Park KI, Bak SB, Lee S, Bae SJ, Kim MJ, et al. Evaluating current status of network pharmacology for herbal medicine focusing on identifying mechanisms and therapeutic effects. J Adv Res. 2024;76:799–815. 39730024 10.1016/j.jare.2024.12.040 PMC12793800

[19] Noor AAM. Exploring the therapeutic potential of terpenoids for depression and anxiety. Chem Biodivers. 2024;21:e202400788. 38934531 10.1002/cbdv.202400788

[20] Xu B, Bai L, Chen L, Tong R, Feng Y, Shi J. Terpenoid natural products exert neuroprotection via the PI3K/Akt pathway. Front Pharmacol. 2022;13:1036506. 36313360 10.3389/fphar.2022.1036506 PMC9606746

[21] Kim JS, Kim JH, Eo H, Ju IG, Son SR, Kim JW, et al. Inulae flos has anti-depressive effects by suppressing neuroinflammation and recovering dysfunction of HPA-axis. Mol Neurobiol. 2024;61:8038–50. 38457106 10.1007/s12035-024-04094-8

[22] Aydin T, Saglamtas R, Dogan B, Kostekci E, Durmus R, Cakir A. A new specific method for isolation of tomentosin with a high yield from Inula viscosa (L.) and determination of its bioactivities. Phytochem Anal. 2022;33:612–8. 35243708 10.1002/pca.3114

[23] He J, Wu H, Zhou Y, Zheng C. Tomentosin inhibit cerebral ischemia/reperfusion induced inflammatory response via TLR4/ NLRP3 signalling pathway - in vivo and in vitro studies. Biomed Pharmacother. 2020;131:110697. 32919189 10.1016/j.biopha.2020.110697

[24] Zaki M, Loubidi M, Bilgic T, Birim D, Akssira M, Dagci T, et al. Design, synthesis, and biological evaluation of novel tomentosin derivatives in NMDA-Induced Excitotoxicity. Pharmaceuticals (Basel). 2022;15:421. 35455419 10.3390/ph15040421 PMC9027110

[25] Talebi M, Khoramjouy M, Feizi A, Ali Z, Khan I, Ayatollahi NA et al. Novel multi-target therapeutic potential of the genus inula: advances and opportunities for neuroprotection. *Pharmacol Res Mod Chin Med* 2023; **7**. 10.1016/j.prmcm.2023.100263.

[26] Zeng T, Liu Z, Zhuang J, Jiang Y, He W, Diao H, et al. TeroKit: a database-driven web server for terpenome research. J Chem Inf Model. 2020;60:2082–90. 32286817 10.1021/acs.jcim.0c00141

[27] Bickerton GR, Paolini GV, Besnard J, Muresan S, Hopkins AL. Quantifying the chemical beauty of drugs. Nat Chem. 2012;4:90–98. 22270643 10.1038/nchem.1243 PMC3524573

[28] Ruiz C, Zitnik M, Leskovec J. Identification of disease treatment mechanisms through the multiscale interactome. Nat Commun. 2021;12:1796. 33741907 10.1038/s41467-021-21770-8 PMC7979814

[29] McIntyre RS, Alsuwaidan M, Baune BT, Berk M, Demyttenaere K, Goldberg JF, et al. Treatment-resistant depression: definition, prevalence, detection, management, and investigational interventions. World Psychiatry. 2023;22:394–412. 37713549 10.1002/wps.21120 PMC10503923

[30] Penn E, Tracy DK. The drugs don’t work? antidepressants and the current and future pharmacological management of depression. Ther Adv Psychopharmacol. 2012;2:179–88. 23983973 10.1177/2045125312445469 PMC3736946

[31] Moncrieff J, Cooper RE, Stockmann T, Amendola S, Hengartner MP, Horowitz MA. The serotonin theory of depression: a systematic umbrella review of the evidence. Mol Psychiatry. 2023;28:3243–56. 35854107 10.1038/s41380-022-01661-0 PMC10618090

[32] Ananth MR, DeLorenzo C, Yang J, Mann JJ, Parsey RV. Decreased pretreatment amygdalae serotonin transporter binding in unipolar depression remitters: a prospective PET study. J Nucl Med. 2018;59:665–70. 28935838 10.2967/jnumed.117.189654 PMC5932749

[33] Guo B, Zhang M, Hao W, Wang Y, Zhang T, Liu C. Neuroinflammation mechanisms of neuromodulation therapies for anxiety and depression. Transl Psychiatry. 2023;13:5. 36624089 10.1038/s41398-022-02297-y PMC9829236

[34] Wang H, He Y, Sun Z, Ren S, Liu M, Wang G, et al. Microglia in depression: an overview of microglia in the pathogenesis and treatment of depression. J Neuroinflammation. 2022;19:132. 35668399 10.1186/s12974-022-02492-0 PMC9168645

[35] Almutabagani LF, Almanqour RA, Alsabhan JF, Alhossan AM, Alamin MA, Alrajeh HM, et al. Inflammation and treatment-resistant depression from clinical to animal study: a possible link?. Neurol Int. 2023;15:100–20. 36648973 10.3390/neurolint15010009 PMC9844360

[36] Attwells S, Setiawan E, Rusjan PM, Xu C, Kish SJ, Vasdev N, et al. A double-blind placebo-controlled trial of minocycline on translocator protein distribution volume in treatment-resistant major depressive disorder. Transl Psychiatry. 2021;11:334. 34052828 10.1038/s41398-021-01450-3 PMC8164633

[37] Haroon E, Daguanno AW, Woolwine BJ, Goldsmith DR, Baer WM, Wommack EC, et al. Antidepressant treatment resistance is associated with increased inflammatory markers in patients with major depressive disorder. Psychoneuroendocrinology. 2018;95:43–49. 29800779 10.1016/j.psyneuen.2018.05.026 PMC6427066

[38] Verdonk F, Roux P, Flamant P, Fiette L, Bozza FA, Simard S, et al. Phenotypic clustering: a novel method for microglial morphology analysis. J Neuroinflammation. 2016;13:153. 27317566 10.1186/s12974-016-0614-7 PMC4912769

[39] Stowell RD, Wong EL, Batchelor HN, Mendes MS, Lamantia CE, Whitelaw BS, et al. Cerebellar microglia are dynamically unique and survey Purkinje neurons in vivo. Dev Neurobiol. 2018;78:627–44. 29285893 10.1002/dneu.22572 PMC6544048

[40] Hinwood M, Morandini J, Day TA, Walker FR. Evidence that microglia mediate the neurobiological effects of chronic psychological stress on the medial prefrontal cortex. Cereb Cortex. 2012;22:1442–54. 21878486 10.1093/cercor/bhr229

[41] Torres-Platas SG, Cruceanu C, Chen GG, Turecki G, Mechawar N. Evidence for increased microglial priming and macrophage recruitment in the dorsal anterior cingulate white matter of depressed suicides. Brain Behav Immun. 2014;42:50–59. 24858659 10.1016/j.bbi.2014.05.007

[42] Bennabi D, Haffen E. Immunomodulation of resistant depression. In: Berk M, Leboyer M, Sommer IE, editors. Immuno-psychiatry: facts and prospects. Cham: Springer International Publishing; 2021. p. 389–400.

[43] Kim YK, Na KS. Role of glutamate receptors and glial cells in the pathophysiology of treatment-resistant depression. Prog Neuropsychopharmacol Biol Psychiatry. 2016;70:117–26. 27046518 10.1016/j.pnpbp.2016.03.009

[44] Enache D, Pariante CM, Mondelli V. Markers of central inflammation in major depressive disorder: a systematic review and meta-analysis of studies examining cerebrospinal fluid, positron emission tomography and post-mortem brain tissue. Brain Behav Immun. 2019;81:24–40. 31195092 10.1016/j.bbi.2019.06.015

[45] Han KM, Ham BJ. How inflammation affects the brain in depression: a review of functional and structural MRI studies. J Clin Neurol. 2021;17:503–15. 34595858 10.3988/jcn.2021.17.4.503 PMC8490908

[46] Kotoula V, Evans JW, Punturieri C, Johnson SC, Zarate CA Jr. Functional MRI markers for treatment-resistant depression: insights and challenges. Progress in Brain Research. 2023;278:117–48. 37414490 10.1016/bs.pbr.2023.04.001 PMC10501192

[47] Pizzagalli DA, Roberts AC. Prefrontal cortex and depression. Neuropsychopharmacology. 2022;47:225–46. 34341498 10.1038/s41386-021-01101-7 PMC8617037

[48] Schnieder TP, Trencevska I, Rosoklija G, Stankov A, Mann JJ, Smiley J, et al. Microglia of prefrontal white matter in suicide. J Neuropathol Exp Neurol. 2014;73:880–90. 25101704 10.1097/NEN.0000000000000107 PMC4141011

[49] Etkin A, Egner T, Kalisch R. Emotional processing in anterior cingulate and medial prefrontal cortex. Trends Cogn Sci. 2011;15:85–93. 21167765 10.1016/j.tics.2010.11.004 PMC3035157

[50] Ge R, Downar J, Blumberger DM, Daskalakis ZJ, Vila-Rodriguez F. Functional connectivity of the anterior cingulate cortex predicts treatment outcome for rTMS in treatment-resistant depression at 3-month follow-up. Brain Stimul. 2020;13:206–14. 31668646 10.1016/j.brs.2019.10.012

[51] Pu S, Nakagome K, Yamada T, Yokoyama K, Matsumura H, Yamada S, et al. Suicidal ideation is associated with reduced prefrontal activation during a verbal fluency task in patients with major depressive disorder. J Affect Disord. 2015;181:9–17. 25913539 10.1016/j.jad.2015.04.010

[52] Pandey GN, Zhang H, Sharma A, Ren X. Innate immunity receptors in depression and suicide: upregulated NOD-like receptors containing pyrin (NLRPs) and hyperactive inflammasomes in the postmortem brains of people who were depressed and died by suicide. J Psychiatry Neurosci. 2021;46:E538–E547. 34588173 10.1503/jpn.210016 PMC8526128

[53] Gustafsson TT, Taipale H, Lahteenvuo M, Tanskanen A, Svirskis T, Huoponen S, et al. Cause-specific mortality in treatment-resistant major depression: population-based cohort study. J Affect Disord. 2025;368:136–42. 39271071 10.1016/j.jad.2024.09.064

[54] Rathod S, Denee T, Eva J, Kerr C, Jacobsen N, Desai M, et al. Health-related quality of life burden associated with treatment-resistant depression in UK patients: quantitative results from a mixed-methods non-interventional study. J Affect Disord. 2022;300:551–62. 34965398 10.1016/j.jad.2021.12.090

[55] Xia CY, Guo YX, Lian WW, Yan Y, Ma BZ, Cheng YC, et al. The NLRP3 inflammasome in depression: potential mechanisms and therapies. Pharmacol Res. 2023;187:106625. 36563870 10.1016/j.phrs.2022.106625

[56] Han KM, Choi KW, Kim A, Kang W, Kang Y, Tae WS, et al. Association of DNA methylation of the NLRP3 gene with changes in cortical thickness in major depressive disorder. Int J Mol Sci. 2022;23:5768. 35628578 10.3390/ijms23105768 PMC9143533

[57] Deng Z, Liu J, He S, Gao W. The pyroptosis-related signature predicts diagnosis and indicates immune characteristic in major depressive disorder. Front Pharmacol. 2022;13:848939. 35677442 10.3389/fphar.2022.848939 PMC9169094

[58] Cattaneo A, Ferrari C, Turner L, Mariani N, Enache D, Hastings C, et al. Whole-blood expression of inflammasome- and glucocorticoid-related mRNAs correctly separates treatment-resistant depressed patients from drug-free and responsive patients in the BIODEP study. Transl Psychiatry. 2020;10:232. 32699209 10.1038/s41398-020-00874-7 PMC7376244

[59] Li S, Fang Y, Zhang Y, Song M, Zhang X, Ding X, et al. Microglial NLRP3 inflammasome activates neurotoxic astrocytes in depression-like mice. Cell Rep. 2022;41:111532. 36288697 10.1016/j.celrep.2022.111532

[60] Wu X, Wan T, Gao X, Fu M, Duan Y, Shen X, et al. Microglia pyroptosis: a candidate target for neurological diseases treatment. Front Neurosci. 2022;16:922331. 35937897 10.3389/fnins.2022.922331 PMC9354884

[61] Liu Z, Wang C, Yang J, Chen Y, Zhou B, Abbott DW, et al. Caspase-1 engages full-length gasdermin D through two distinct interfaces that mediate caspase recruitment and substrate cleavage. Immunity. 2020;53:106–114.e5. 32553275 10.1016/j.immuni.2020.06.007 PMC7382298

[62] Yang J, Liu Z, Wang C, Yang R, Rathkey JK, Pinkard OW, et al. Mechanism of gasdermin D recognition by inflammatory caspases and their inhibition by a gasdermin D-derived peptide inhibitor. Proc Natl Acad Sci USA. 2018;115:6792–7. 29891674 10.1073/pnas.1800562115 PMC6042100

[63] Masyita A, Mustika Sari R, Dwi Astuti A, Yasir B, Rahma Rumata N, Emran TB, et al. Terpenes and terpenoids as main bioactive compounds of essential oils, their roles in human health and potential application as natural food preservatives. Food Chem X. 2022;13:100217. 35498985 10.1016/j.fochx.2022.100217 PMC9039924

[64] Vora LK, Gholap AD, Hatvate NT, Naren P, Khan S, Chavda VP, et al. Essential oils for clinical aromatherapy: a comprehensive review. J Ethnopharmacol. 2024;330:118180. 38614262 10.1016/j.jep.2024.118180

[65] Mony TJ, Elahi F, Choi JW, Park SJ. Neuropharmacological effects of terpenoids on preclinical animal models of psychiatric disorders: a review. Antioxidants (Basel). 2022;11:1834. 36139909 10.3390/antiox11091834 PMC9495487

[66] de Lima EP, Laurindo LF, Catharin VCS, Direito R, Tanaka M, Jasmin Santos German I, et al. Polyphenols, alkaloids, and terpenoids against neurodegeneration: evaluating the neuroprotective effects of phytocompounds through a comprehensive review of the current evidence. Metabolites. 2025;15:124. 39997749 10.3390/metabo15020124 PMC11857241

[67] Spindle TR, Zamarripa CA, Russo E, Pollak L, Bigelow G, Ward AM, et al. Vaporized D-limonene selectively mitigates the acute anxiogenic effects of Delta9-tetrahydrocannabinol in healthy adults who intermittently use cannabis. Drug Alcohol Depend. 2024;257:111267. 38498958 10.1016/j.drugalcdep.2024.111267 PMC11031290

[68] Huang W, Wang Y, Tian W, Cui X, Tu P, Li J, et al. Biosynthesis investigations of terpenoid, alkaloid, and flavonoid antimicrobial agents derived from medicinal plants. Antibiotics (Basel). 2022;11:1380. 36290037 10.3390/antibiotics11101380 PMC9598646

[69] Lautie E, Russo O, Ducrot P, Boutin JA. Unraveling plant natural chemical diversity for drug discovery purposes. Front Pharmacol. 2020;11:397. 32317969 10.3389/fphar.2020.00397 PMC7154113

[70] Noori T, Sureda A, Sobarzo-Sanchez E, Shirooie S. The role of natural products in treatment of depressive disorder. Curr Neuropharmacol. 2022;20:929–49. 34979889 10.2174/1570159X20666220103140834 PMC9881107

[71] Turner EH. Esketamine for treatment-resistant depression: seven concerns about efficacy and FDA approval. Lancet Psychiatry. 2019;6:977–9. 31680014 10.1016/S2215-0366(19)30394-3

[72] Pereira S, Brennan E, Patel A, Moran M, Wallier J, Liebowitz MR. Managing dissociative symptoms following the use of esketamine nasal spray: a case report. Int Clin Psychopharmacol. 2021;36:54–57. 32804743 10.1097/YIC.0000000000000327 PMC7690636

[73] Hellmann-Regen J, Clemens V, Grozinger M, Kornhuber J, Reif A, Prvulovic D, et al. Effect of minocycline on depressive symptoms in patients with treatment-resistant depression: a randomized clinical trial. JAMA Netw Open. 2022;5:e2230367. 36103181 10.1001/jamanetworkopen.2022.30367 PMC9475381

[74] Shamim MA, Manna S, Dwivedi P, Swami MK, Sahoo S, Shukla R, et al. Minocycline in depression not responding to first-line therapy: a systematic review and meta-analysis. Medicine (Baltimore). 2023;102:e35937. 37960804 10.1097/MD.0000000000035937 PMC10637431

