# Supplementary material for: Tomentosin selectively targets microglial pyroptosis to overcome fluoxetine-resistant depression: a network-based therapeutic discovery
Source: Transl Psychiatry. 2026 May 15;16:346. doi: 10.1038/s41398-026-04092-5 (PMC13347013; doi:10.1038/s41398-026-04092-5)

**Supplementary Materials**

**Material and Methods**

*Tail suspension test.* Depressive-like behavior was examined using a large square box (50 × 30 × 55 cm). Each mouse was individually suspended by the tail from the hook with an adhesive tape and allowed to hang for 6 min. The latency time was measured when a mouse initially adopted an immobile position, which is defined as a mouse that stops in a stay position after 1 minute and makes only small movements to hang. The number of times a mouse assumed a mobile position was recorded for 5 min.

*Forced swimming test.* Despair behavior was examined using a cylindrical container (20 × 30 cm). Then the container was ﬁlled with water (24 ± 1 °C) to a depth of 20 cm. The mice were individually placed in each container and allowed to swim for 6 min. Latency time was recorded when a mouse ﬁrst assumed an immobile position, deﬁned as a mouse ﬂoating in an upright position after 1 min, making only small movements to keep its head above water. The number of times a mouse assumed a mobile position was recorded for 5 min.

*Nest building test.* Sickness-like behavior was evaluated using pressed cotton squares (Envigo, IN, USA). Briefly, a total of 12 g of pressed cotton squares (ten squares per cage, 7 × 5 cm) were positioned in the center of the floor of a cage that housed eight mice. The mice were scored on a scale of 0 to 5 based on the extent to which they bit the squares, moved them into the corners, and nested with the squares overnight.

*Open field test*. Anxious behavior was evaluated using a large square chamber (40 × 40 × 30 cm) where center area (25 × 25 cm) designated in recording software. After acclimation for 30 min in a testing room, the mice were allowed to freely explore the field for 5 min. The time spent in the center area was recorded.

*Marble burying test.* Anxiety-like behavior was evaluated using marbles (Envigo, IN, USA). Briefly, a total of 12 marbles were evenly spaced, in the floor of a cage that housed each mouse. After 30 min, the marbles were counted, which were at least two-thirds of the number covered with sawdust. The test mice were put back into the house cage. The number of marbles that the mouse dug during a 30-min period was evaluated on a scale.


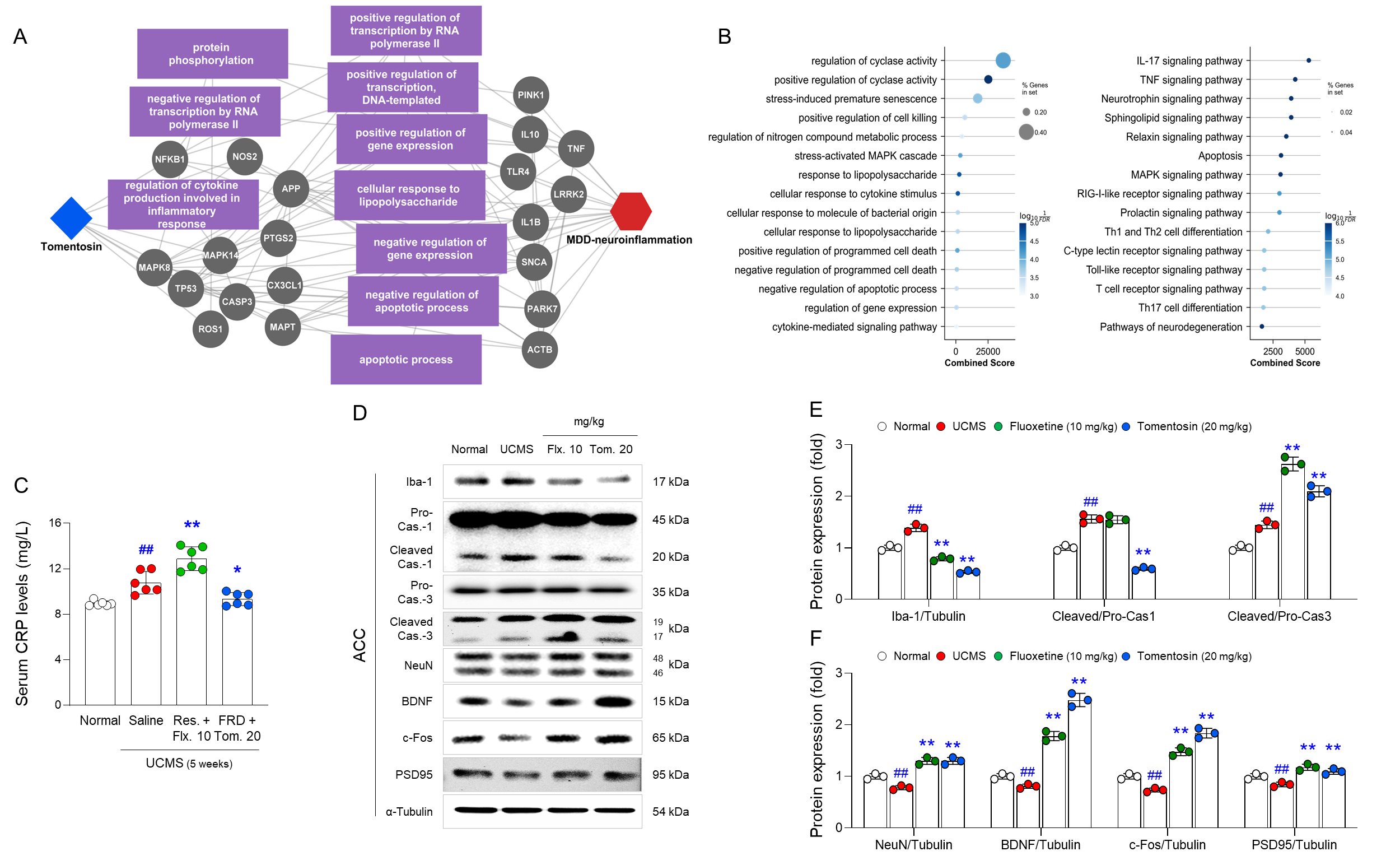


**Supplementary Figure 1. Gene set enrichment analysis and effects of tomentosin on microglial pyroptosis and serum CRP levels.** Gene ontology (A) and KEGG annotation (B) analyses revealed significant associations of tomentosin with key protein targets and relevant biological functions. Serum CRP levels were evaluated by ELISA (C). Protein levels of Iba-1, cleaved/pro caspase-1 ratio, cleaved/pro caspase-3 ratio, NeuN, BDNF, c-Fos and PSD95 expression in ACC homogenates were determined by western blotting analysis (D), and its intensities were semi-quantified (E and F). Data are expressed as the mean ± SD (n = 3 or 6/group). ^##^*p* < 0.01 compared to the normal mice; ^**^*p* < 0.01 compared to the UCMS-subjected mice.


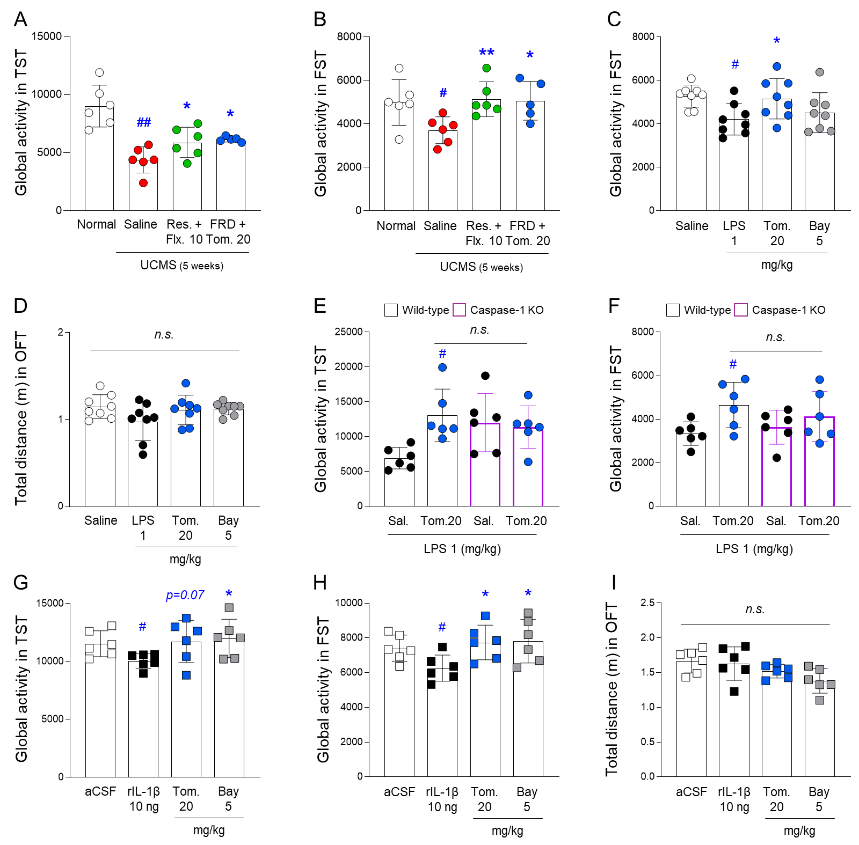


**Supplementary Figure 2. Effects of tomentosin on MDD-like behaviors across multiple mouse models.** In the UCMS model, the global activity in TST (A) and FST (B) were measured. In the systemic LPS-injected model, the global activity in FST (C) and the total distance in OFT (D) were evaluated. In the Casp1 KO model, the global activity in TST (E) and FST (F) were evaluated. In the intracranial rIL-1β-injected model, the global activity in TST (G) and FST (H) along with the total distance in OFT (I) were assessed. Data are expressed as the mean ± SD (n = 6 or 8/group). ^#^*p* < 0.05 and ^##^*p* < 0.01 compared to the corresponding control mice; ^*^*p* < 0.05 and ^**^*p* < 0.01 compared to the corresponding induced mice.


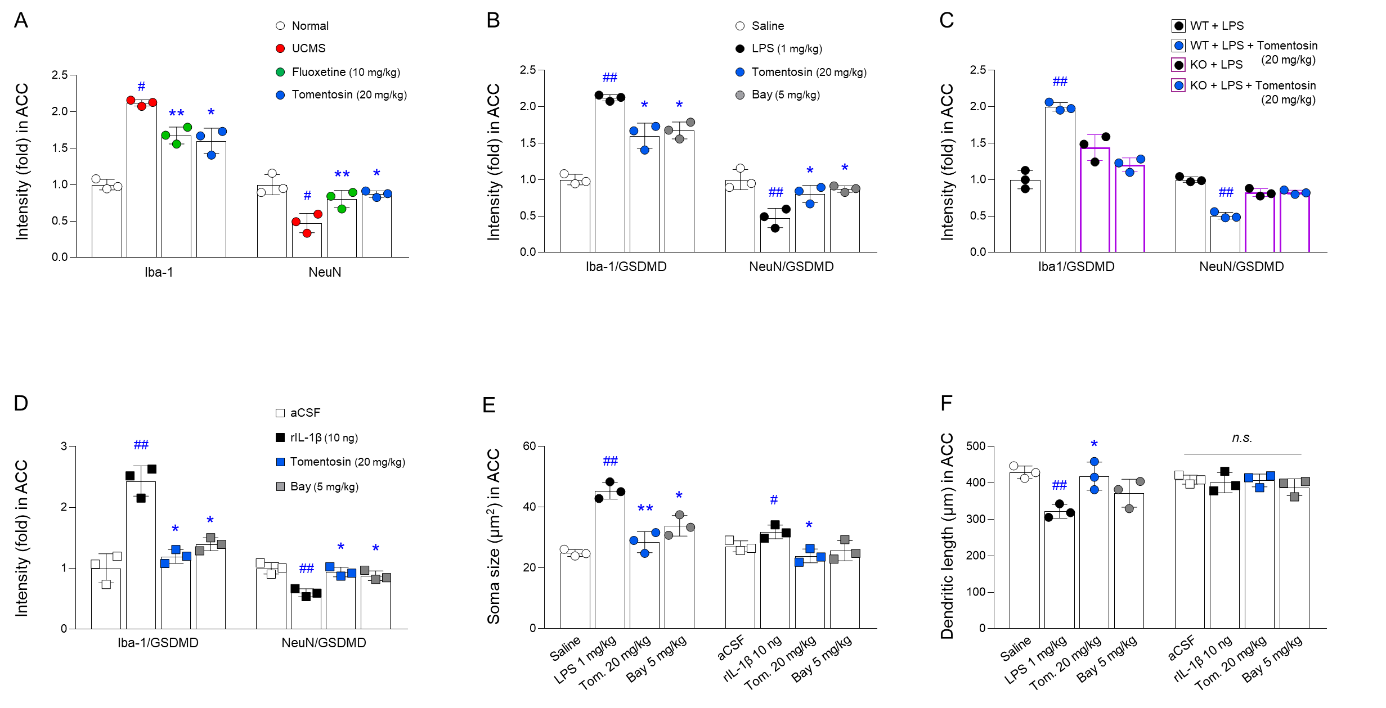


**Supplementary Figure 3. Effects of tomentosin on microglial pyroptosis and morphologic features across serial mouse models.** In the UCMS model, Iba-1 positive signals and NeuN positive signals in the ACC were semi-quantified (A). In the systemic LPS-injected model (B), caspase-1 KO model (C), and intracranial rIL-1β-injected model (D), Iba-1/GSDMD double-positive signals and NeuN positive signals in the ACC were semi-quantified. Additionally, microglial soma size (E) and process length (F) were evaluated across the respective mouse models. Data are expressed as the mean ± SD (n = 3/group). ^#^*p* < 0.05 and ^##^*p* < 0.01 compared to the corresponding control mice; ^*^*p* < 0.05 and ^**^*p* < 0.01 compared to the corresponding induced mice.


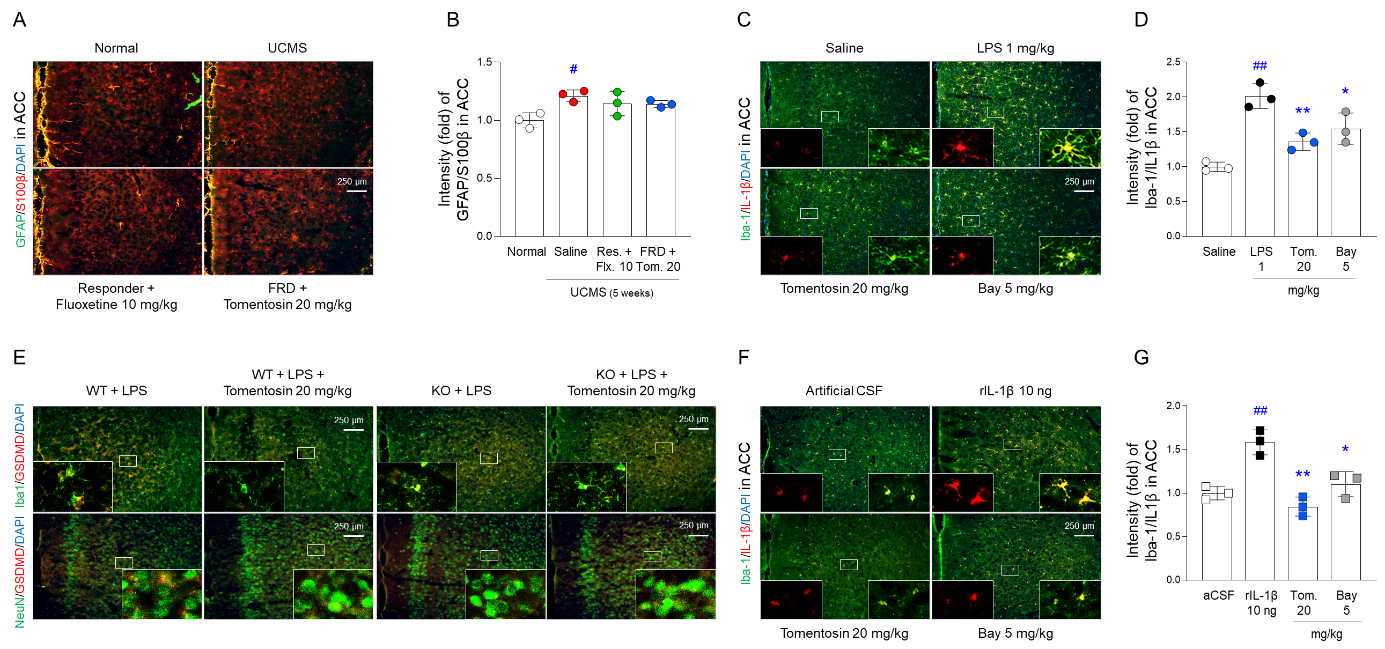


**Supplementary Figure 4. Effects of tomentosin on astrocyte activity and microglial inflammatory signals in serial mouse models.** Representative immunofluorescence images and semi-quantification of GFAP/S100β double-positive signals in the ACC of the UCMS model (A and B). Representative images and semi-quantification of Iba-1/IL-1β double-positive signals in the systemic LPS-injected model (C and D), and the intracranial rIL-1β-injected model (F and G). Representative immunofluorescence images showing Iba-1/GSDMD and NeuN/GSDMD double-positive signaling in the ACC of the Casp1 KO model. (E). Data are expressed as the mean ± SD (n = 3/group). ^#^*p* < 0.05 and ^##^*p* < 0.01 compared to the corresponding control mice; ^*^*p* < 0.05 and ^**^*p* < 0.01 compared to the corresponding induced mice.


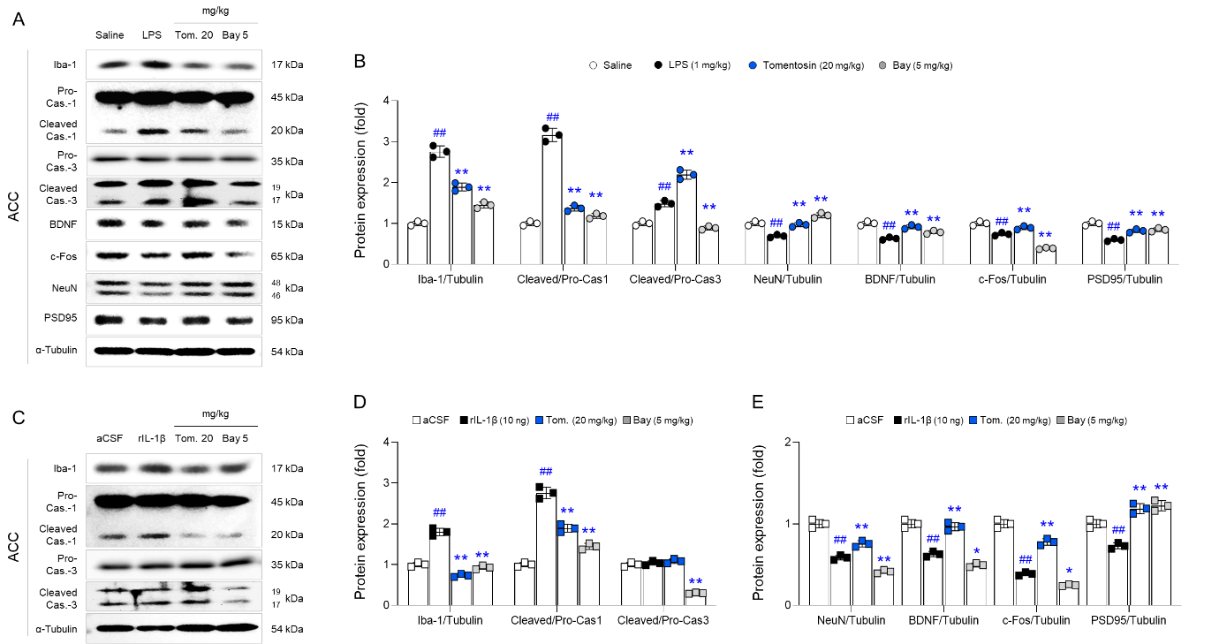


**Supplementary Figure 5. Effects of tomentosin on microglial cell death markers and neuronal activity-related molecules across serial mouse models.** In the ACC, levels of proteins associated with microglial activation (Iba-1), pyroptosis (cleaved/pro-caspase-1), apoptosis (cleaved/pro-caspase-3), and neuronal activity (NeuN, c-Fos, BDNF, and PSD95) were examined by Western blotting analysis in the systemic LPS-injected model (A) and intracranial rIL-1β-injected model (C). The protein levels of these explored molecules in brain tissue lysates were semi-quantified for the respective models (B and D). Additionally, the protein expression of neuronal activity-related markers (NeuN, c-Fos, BDNF, and PSD95) in the rIL-1β model was further quantified (E). Data are expressed as the mean ± SD (n = 3/group). ^##^*p* < 0.01 compared to the corresponding control mice; ^*^*p* < 0.05 and ^**^*p* < 0.01 compared to the corresponding induced mice.


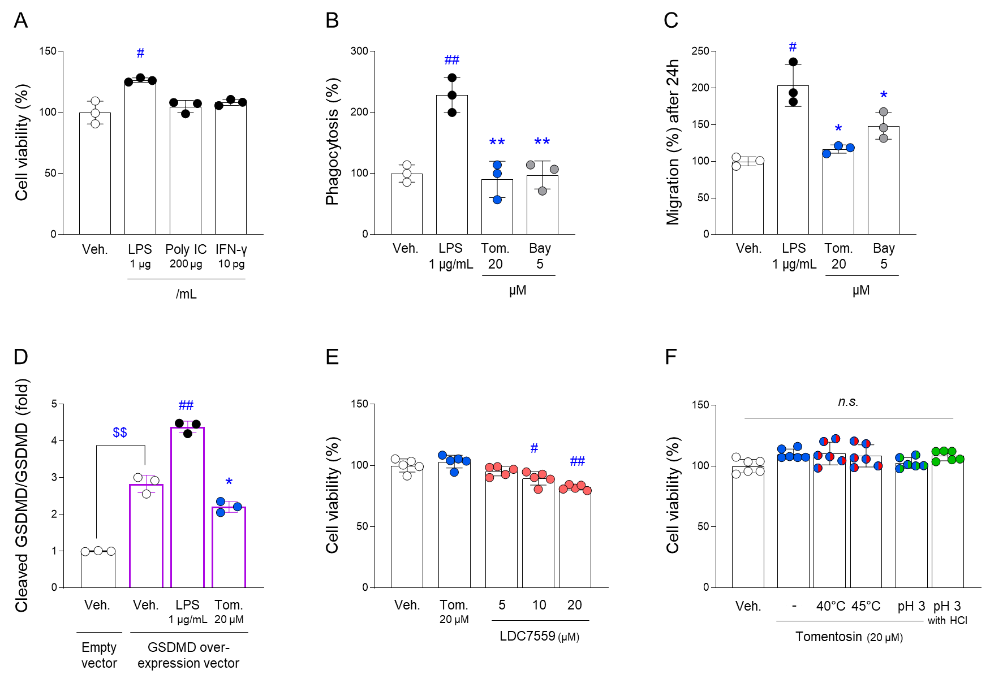


**Supplementary Figure 6. Effects of tomentosin on activated BV2 microglia.** The cytotoxicity of tomentosin and three inducers (LPS, poly I:C, and IFN-γ) in BV2 microglial cells was evaluated by the WST-8 cell viability assay (A). Under LPS exposure, phagocytic activity was assessed using a FITC-fluorescent phagocytosis assay, followed by semi-quantification (B). In response to LPS exposure and/or tomentosin treatment, migratory activity was assessed by measuring the relative changes in migration toward the injury site, followed by semi-quantification (C). Under conditions of GSDMD overexpression and LPS stimulation, the inhibitory effect of tomentosin on GSDMD cleavage was semi-quantified (D; see also Figure 6D for additional quantification). The cytotoxicity of LDC7559 and tomentosin exposed to thermal or acidic changes was measured by the WST-8 cell viability assay (E and F). Data are expressed as the mean ± SD (n = 3, 5 or 6/group). ^$$^*p* < 0.01 compared to the empty vector-transfected cells; ^#^*p* < 0.05 and ^##^*p* < 0.01 compared to the vehicle-treated cells; ^*^*p* < 0.05 and ^**^*p* < 0.01 compared to the stimulator-exposed cells.

**
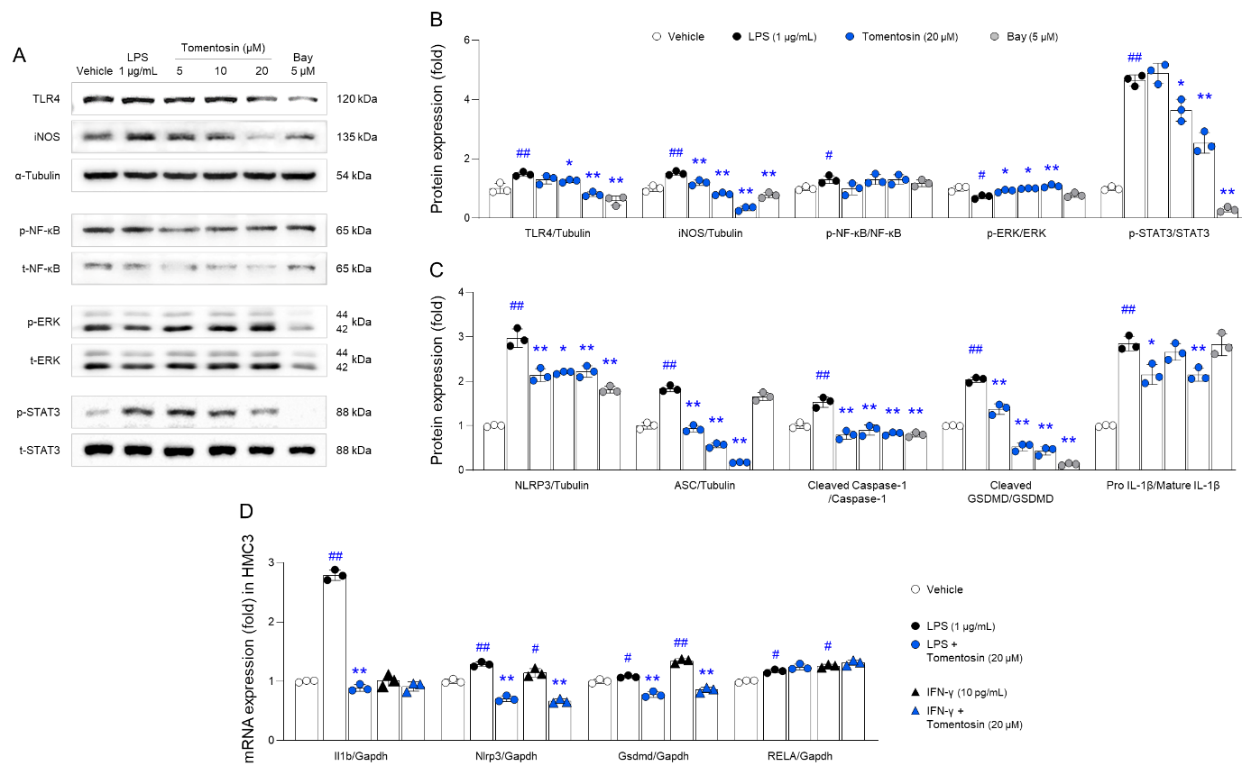
**

**Supplementary Figure 7. Effects of tomentosin on activated BV2 and HMC3 microglial-derived molecular alterations.** In LPS-stimulated BV2 microglia, levels of proteins associated with the TLR4/NF-κB/STAT3 inflammatory pathway were examined by Western blotting analysis (A). The protein levels of these explored molecules in cell lysates were semi-quantified (B and C). In LPS- or IFN-γ exposed HMC3 human microglia, the mRNA expression levels of inflammation and inflammasome genes (*IL1B, NLRP3, GSDMD,* and *RELA*) were measured and semi-quantified by RT-PCR (D). Data are expressed as the mean ± SD (n = 3/group). ^#^*p* < 0.05 and ^##^*p* < 0.01 compared to the vehicle-treated cells; ^*^*p* < 0.05 and ^**^*p* < 0.01 compared to the LPS- or IFN-γ treated cells.

**
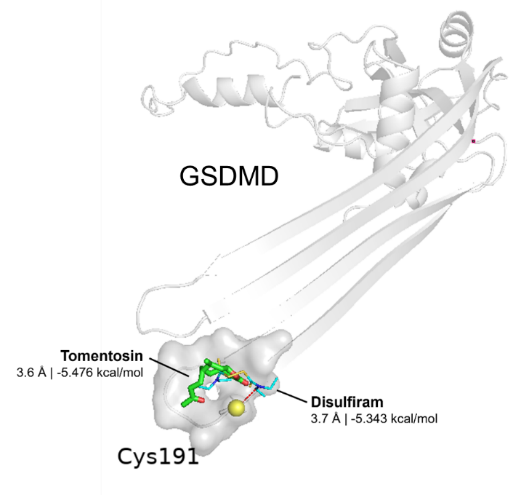
**

**Supplementary Figure 8. Predicted binding mode of tomentosin at the disulfiram-binding site (Cys191) of GSDMD.** Local docking simulations performed on the AlphaFold-predicted GSDMD structure reveal overlapping binding poses for tomentosin (green) and disulfiram (cyan). Both ligands exhibit comparable binding affinities (~-5.4 kcal/mol) and proximity (~3.6–3.7 Å) to the reactive Cys191 residue (yellow sphere).


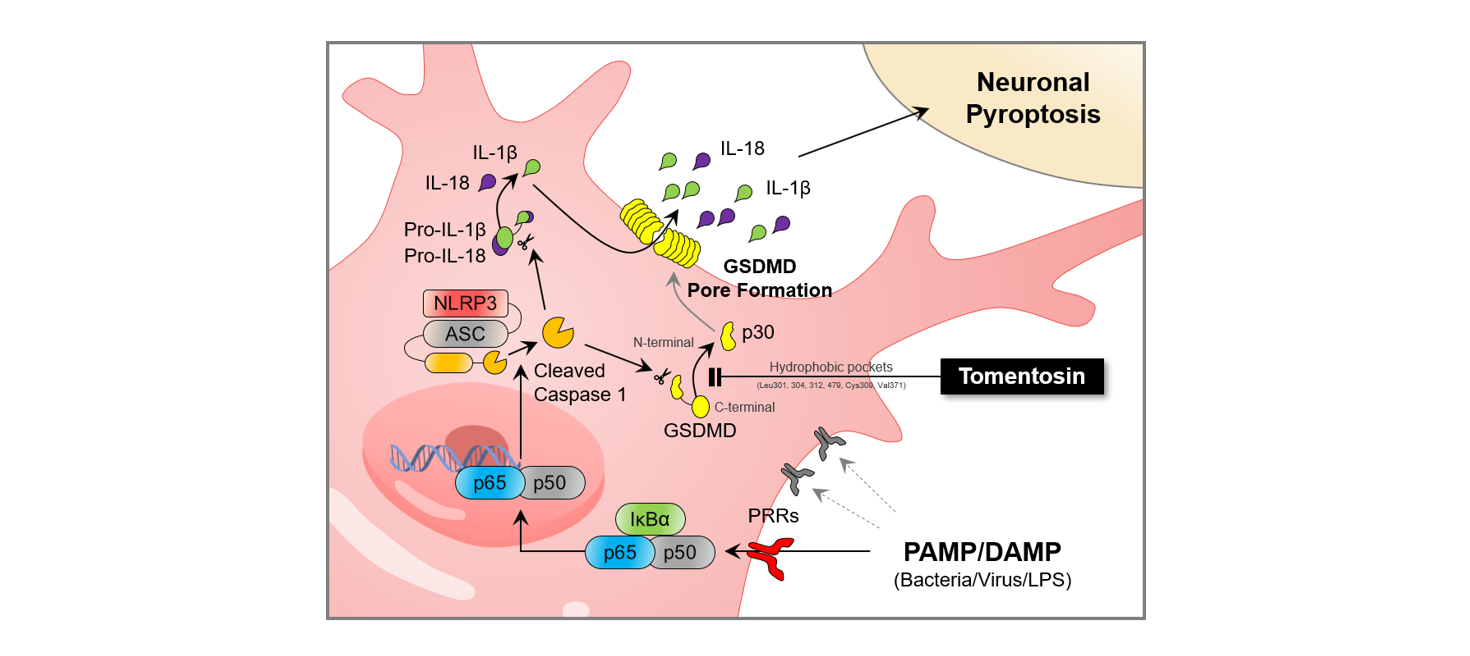


**Supplementary Figure 9.** **Schematic diagram illustration of Tomentosin’s mechanism of action in microglia.**

| Day | UCMS schedule | |
| --- | --- | --- |
| 1 | Forced swim (20 min, 22 ± 2°C) | Restraint stress (6 h) |
| 2 | Wet bedding (24 h with bedding chip 1 : tap water 1) | |
| 3 | Electric foot shock (100 sec, 0.5 mA) | Predator sound (2 h) |
| 4 | Tail fixation (2 h, fixing with medical tape but forepaw on the ground) | |
| 5 | Empty cage (24 h without bedding chip) + Light illusion (24 h) | |
| 6 | Sleep deprivation (12 h, applying modified multiple platform method) | |
| 7 | Tail suspension (2 h, hanging on a pole) | Empty cage |
| 8 | Electric foot shock | Restraint stress |
| 9 | Forced swim | |
| 10 | Sleep deprivation | |
| 11 | Predator sound | Wet bedding |
| 12 | Cage tilt (24 h, 45°) + Light on (24 h) | |
| 13 | Uncomfortable cage (24h with wire bottom) + Food deprivation (24h) | |
| 14 | Sleep deprivation | |
| 15 | Electric foot shock | Predator sound |
| 16 | Tail suspension | Food deprivation |
| 17 | Cage swap (24h, exchanging with rat bedding) | |
| 18 | Restraint stress | Forced swim |
| 19 | Cage tilt + Light on | |
| 20 | Empty cage + Light off (24 h) | |
| 21 | Wet bedding + Electric foot shock | |
| 22 | Forced swim | Restraint stress |
| 23 | Social defeat (10 min, cohabiting with ICR) + Wet bedding | |
| 24 | Electric foot shock | Predator sound |
| 25 | Tail fixation | |
| 26 | Empty cage + Light illusion | |
| 27 | Sleep deprivation | |
| 28 | Tail suspension | Empty cage |
| 29 | Electric foot shock | Restraint stress |
| 30 | Forced swim | |
| 31 | Sleep deprivation | |
| 32 | Predator sound | Wet bedding |
| 33 | Cage tilt + Light on | |
| 34 | Uncomfortable cage + Food deprivation | |
| 35 | Sleep deprivation | |

**Supplementary Table 1.** **Experimental schedules of UCMS**

| Tomentosin (PubChem ID: 155173) | | | | |
| --- | --- | --- | --- | --- |
| Type | Protein | CB-Dock2 affinity (kcal/mol) † | Local bind affinity (kcal/mol) ‡ | Identifier |
| *Mus musculus* | NLRP3 dodecamer | -7.0 | -5.6 | 7VTQ |
|  | IL-1β | -6.2 | -4.8 | 2MIB |
|  | GSDMD | -7.6 | -6.3 | 6N9N |
|  | Caspase-1 | -5.6 | -4.7 | 6VIE* |
|  | STAT3 | -6.6 | -4.8 | 4ZIA |
| *Homo sapiens* | NLRP3 hexamer | -7.9 | -5.8 | 7VTP |
|  | IL-1β | -6.8 | -4.6 | 1ITB* |
|  | GSDMD | -7.7 | -6.1 | 6N9O |
|  | Caspase-1 | -6.9 | -4.2 | 1RWK |
|  | STAT3 | -6.7 | -4.4 | 6NJS |

**Supplementary Table 2.** **Molecular docking affinity with tomentosin**

GSDMD; gasdermin d, IL; interleukin, NLRP; NLR family pyrin domain containing, STAT; signal transducer and activator of transcription.

† CB-Dock2 Affinity: Scores obtained from the CB-Dock2 server.

‡ Local Vina Affinity: Scores obtained from optimized site-specific AutoDock Vina simulations.

* Structure isolated from a larger complex (Caspase-1 from Caspase-1/GSDMD and IL-1R from IL-1R/IL-1β complexes).

**Supplementary Table 3.** Sequence of the primers used in real-time PCR analysis.

| Gene (number) | Primer sequence (Forward and Reverse) |
| --- | --- |
| *IL1B*  (NM_021975.4) | 5'-CCACAGACCTTCCAGGAGAATG-3'  5'-GTGCAGTTCAGTGATCGTACAGG-3' |
| *NLRP3*  (NM_000576.3) | 5'-GGACTGAAGCACCTGTTGTGCA-3'  5'-TCCTGAGTCTCCCAAGGCATTC-3' |
| *GSDMD*  (NM_004895.5) | 5′-ATGAGGTGCCTCCACAACTTCC-3′  5′-CCAGTTCCTTGGAGATGGTCTC-3′ |
| *RELA*  (NM_024736.7) | 5'-GGAGCACAGATACCACCAAGAC-3'  5'-CTCAGCCTCATAGAAGCCATCC-3' |
| *GAPDH*  (NM_002046.7) | 5'-GTCTCCTCTGACTTCAACAGCG-3'  5'-ACCACCCTGTTGCTGTAGCCAA-3' |

Gapdh; Glyceraldehyde 3-phosphate dehydrogenase, Gsdmd; Gasdermin d, Il1b; Interleukin 1beta, Nlrp3; NLR family, pyrin domain containing 3, RELA; Rela proto-oncogene (p65 NF-κB subunit)

**Uncropped Blots images**

**
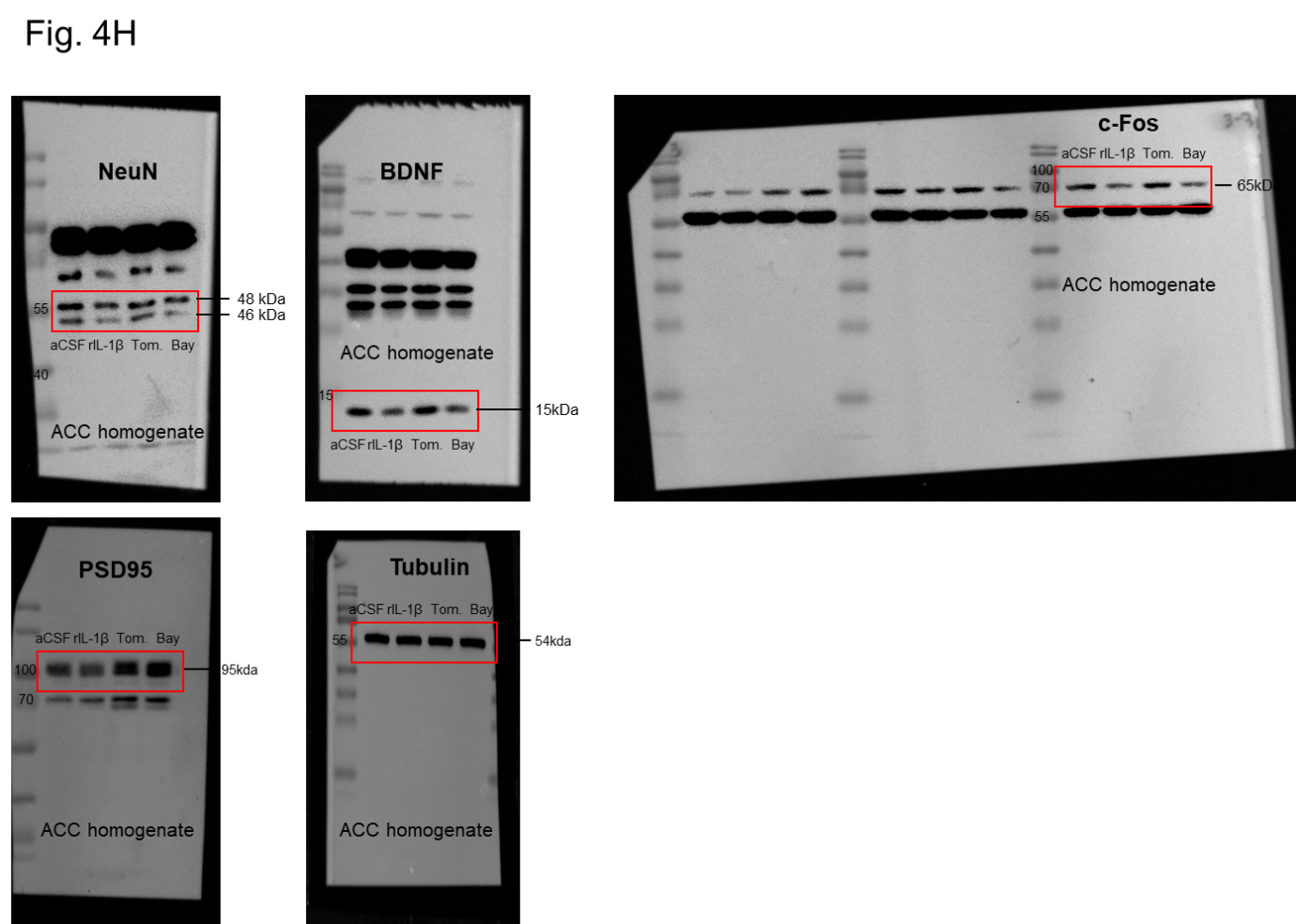

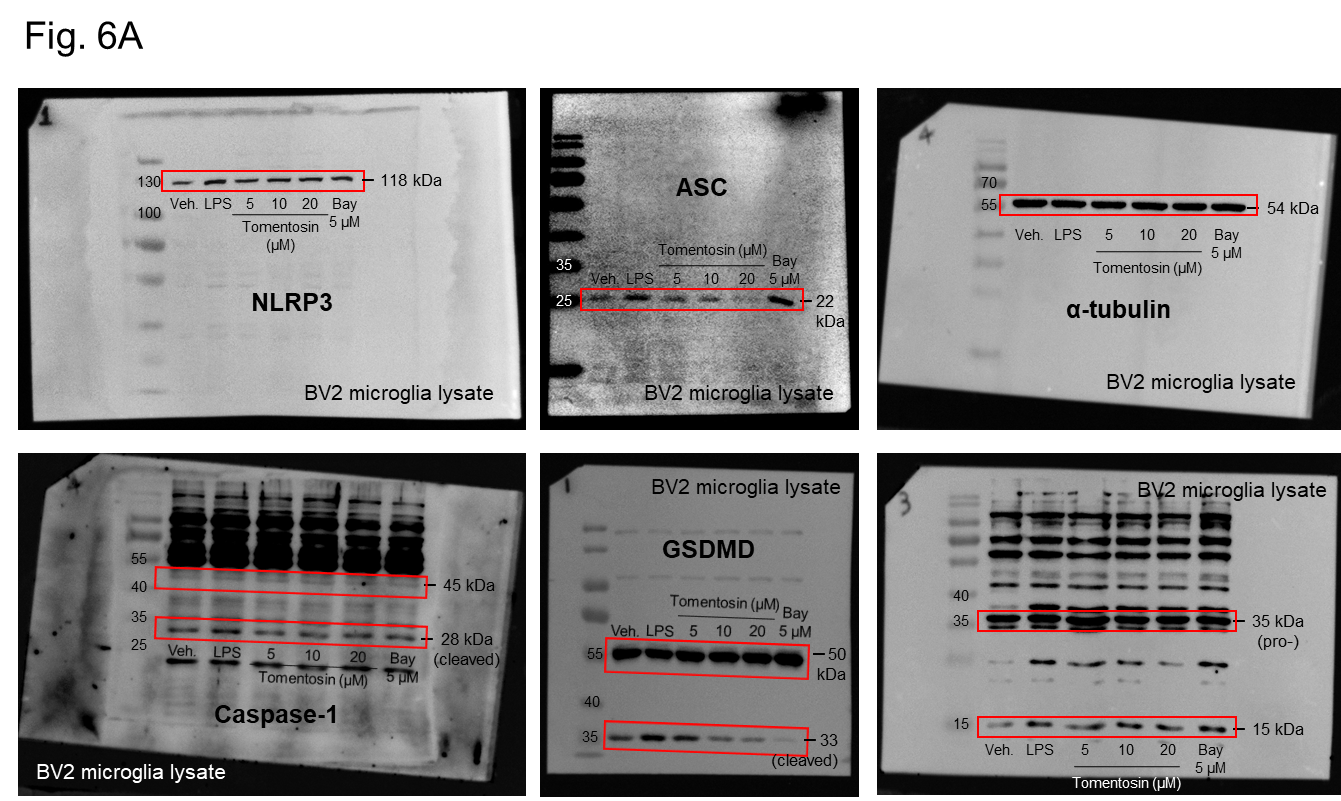

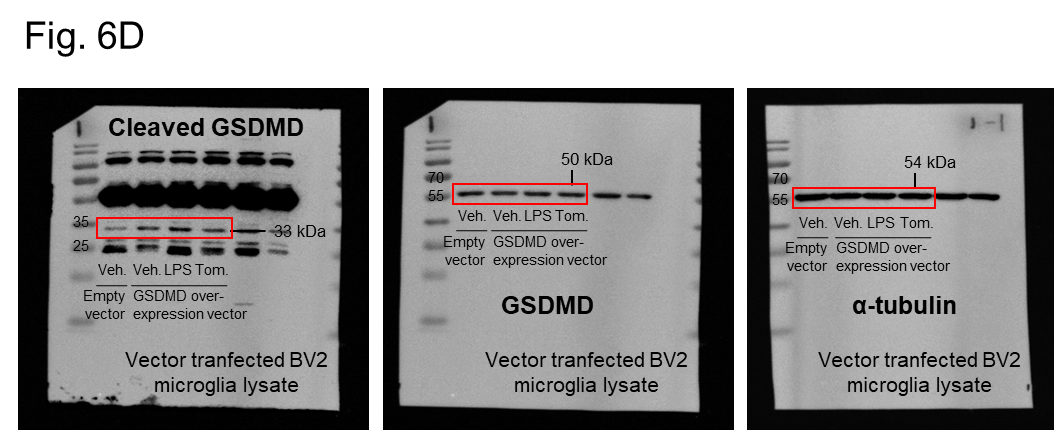
**


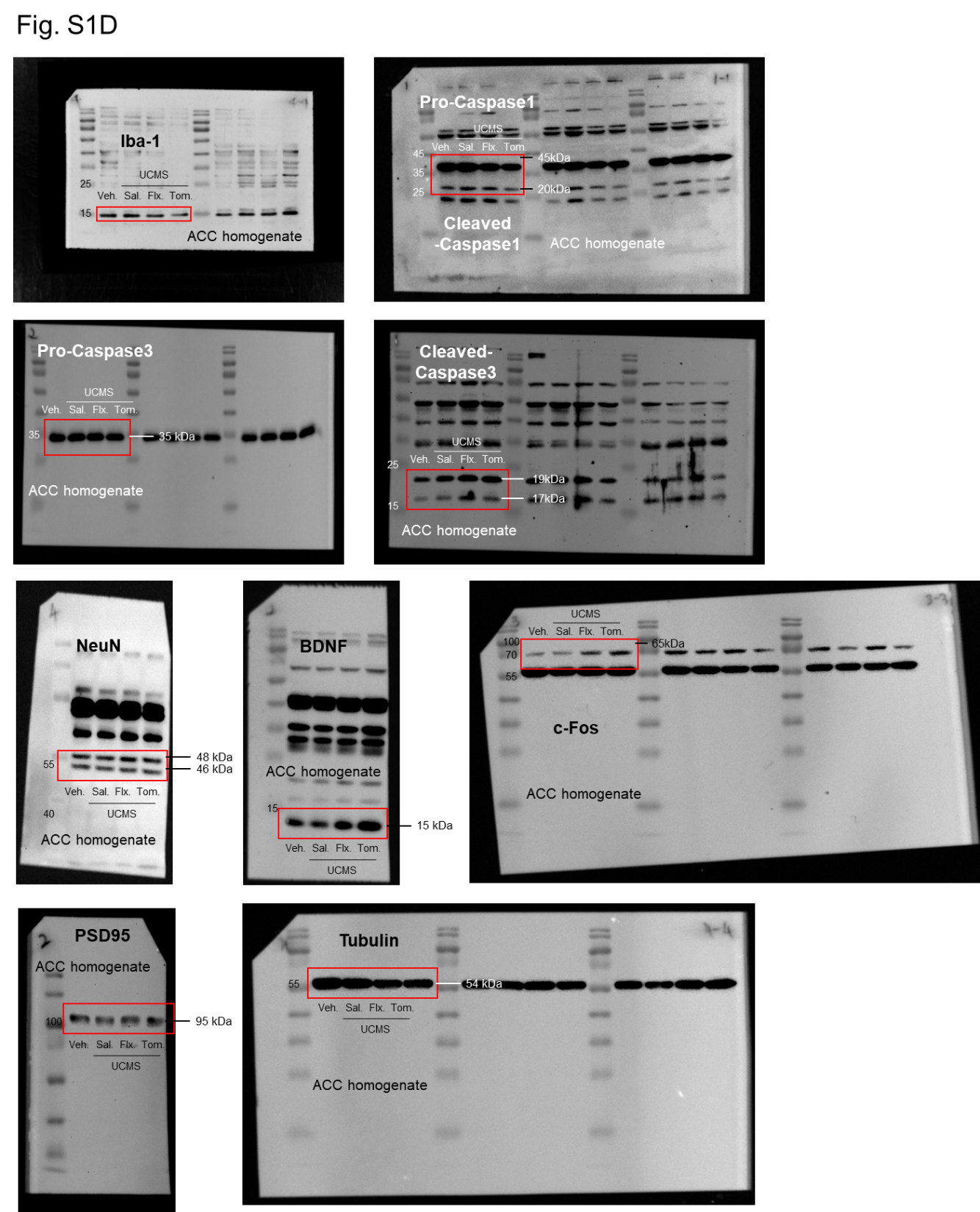


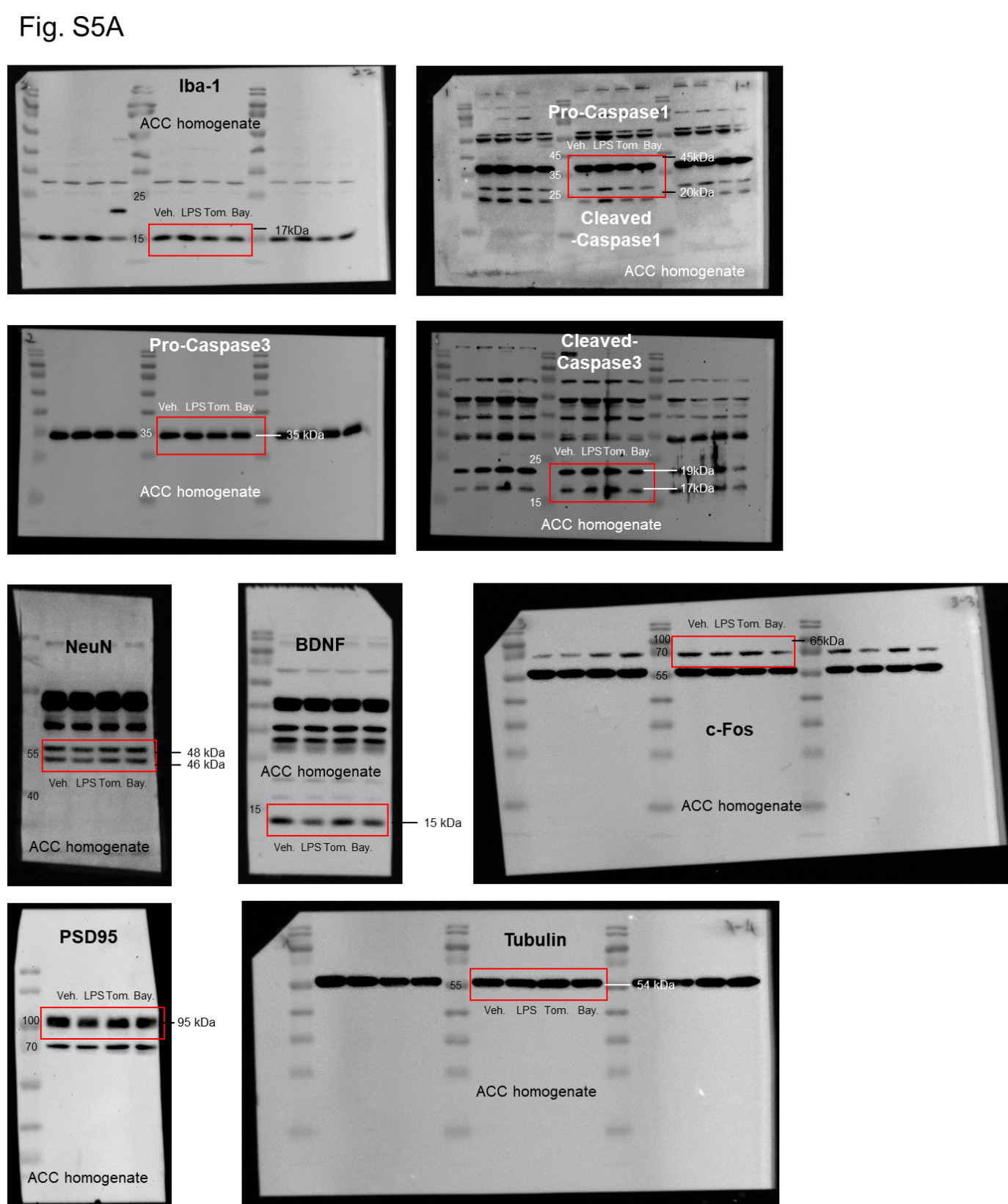

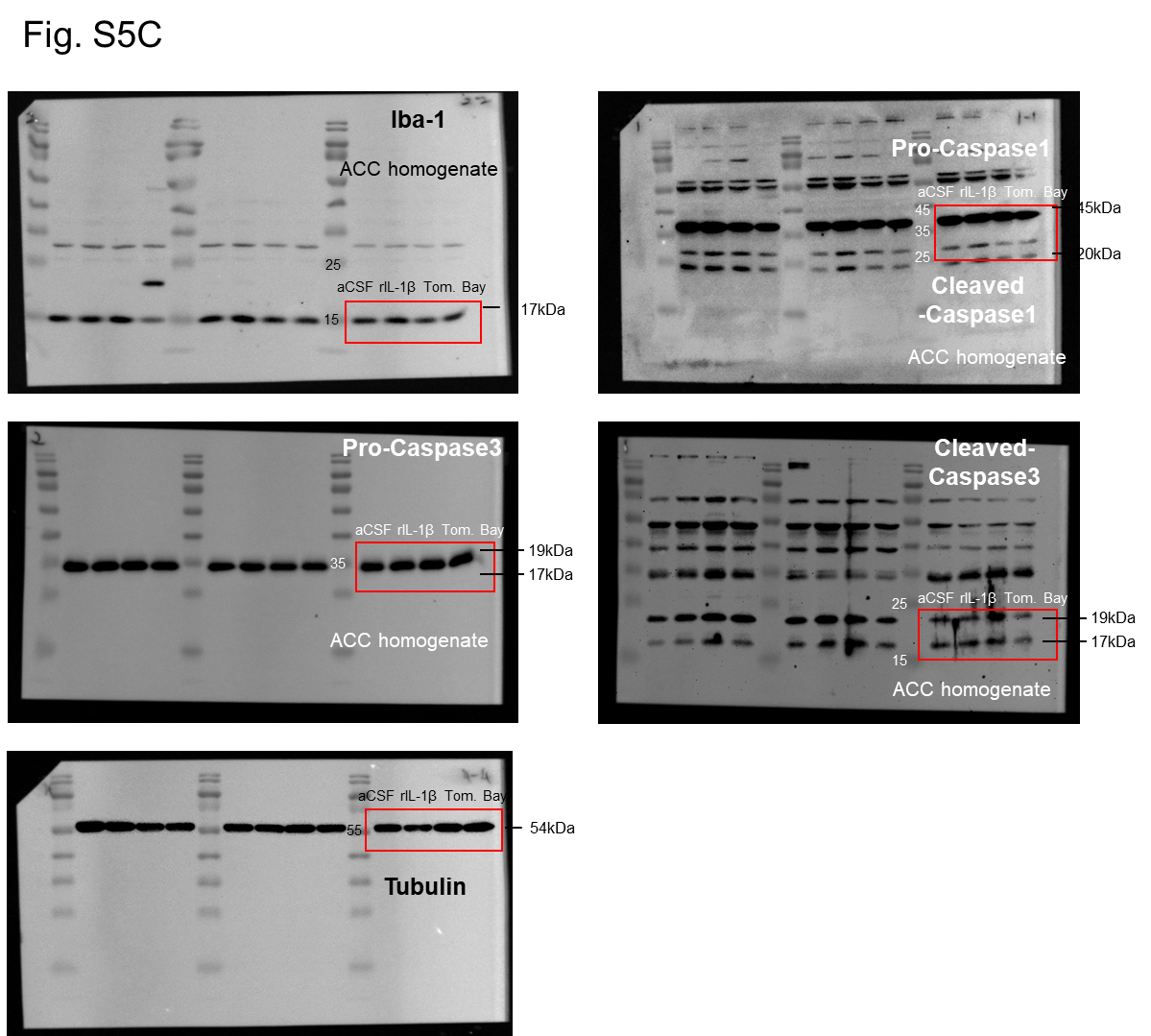


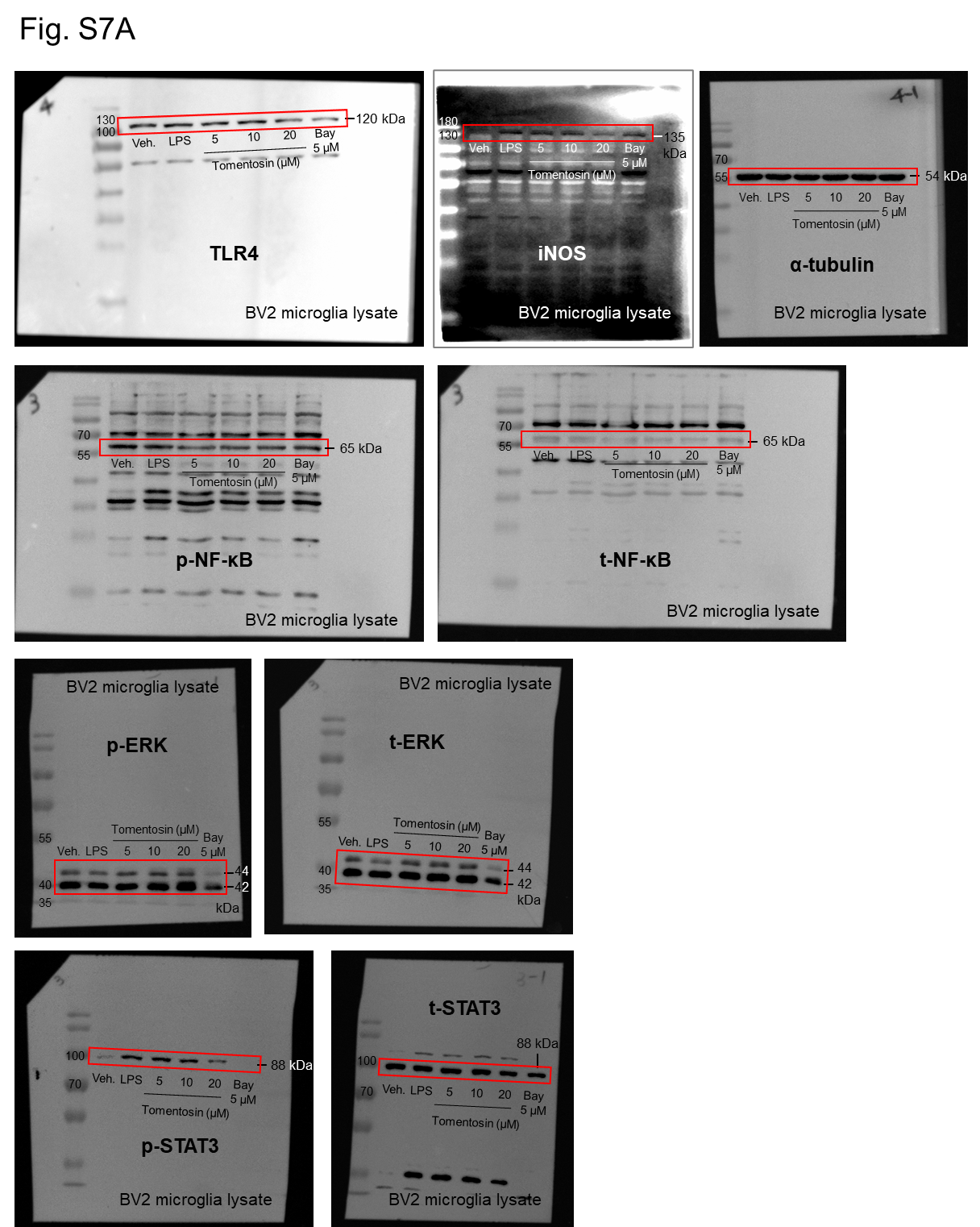


**Original immunofluorescent images**

UCMS model (GFAP-S100β)


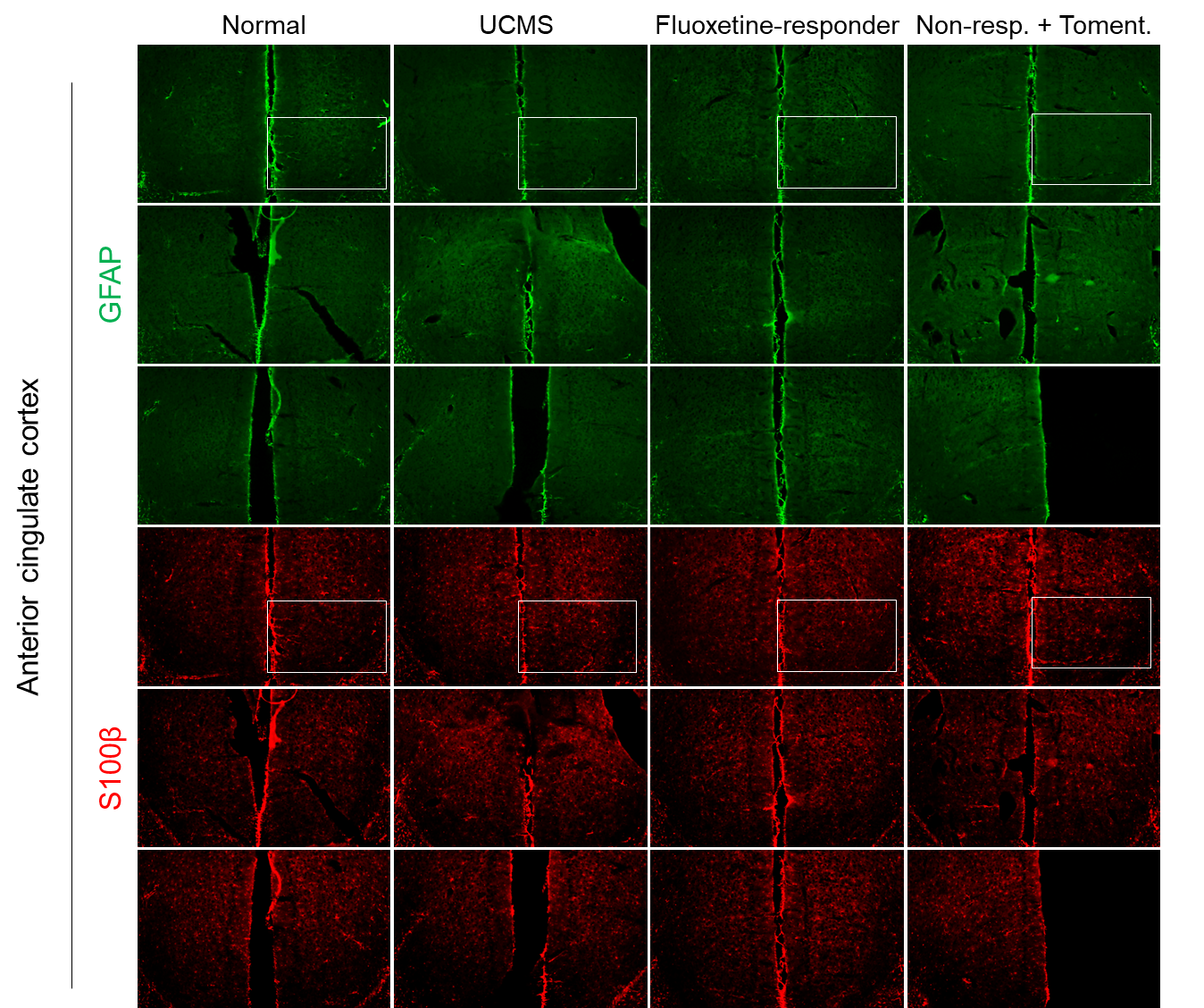


UCMS model (Iba-NeuN)


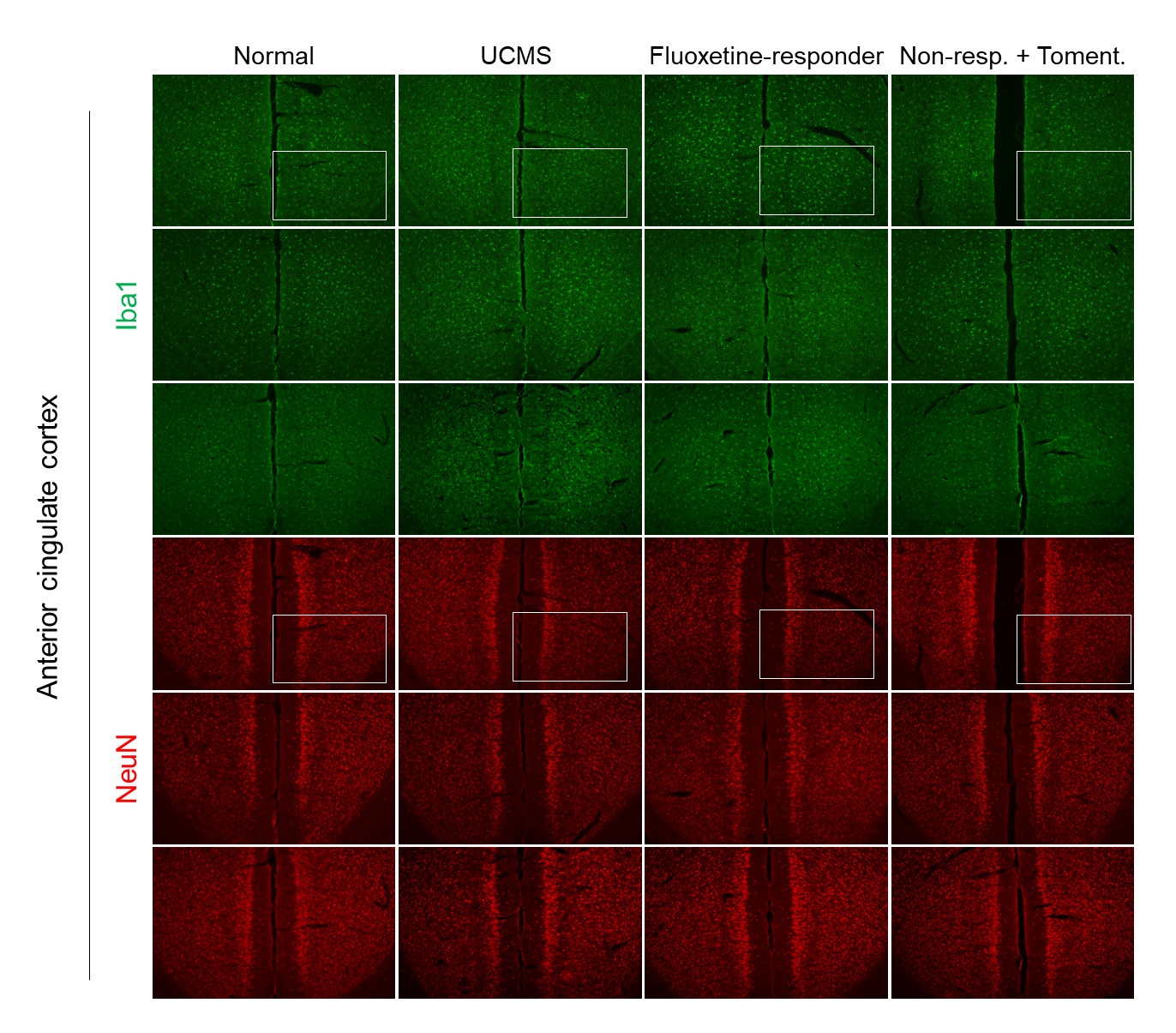


UCMS model (Iba1-IL1b)


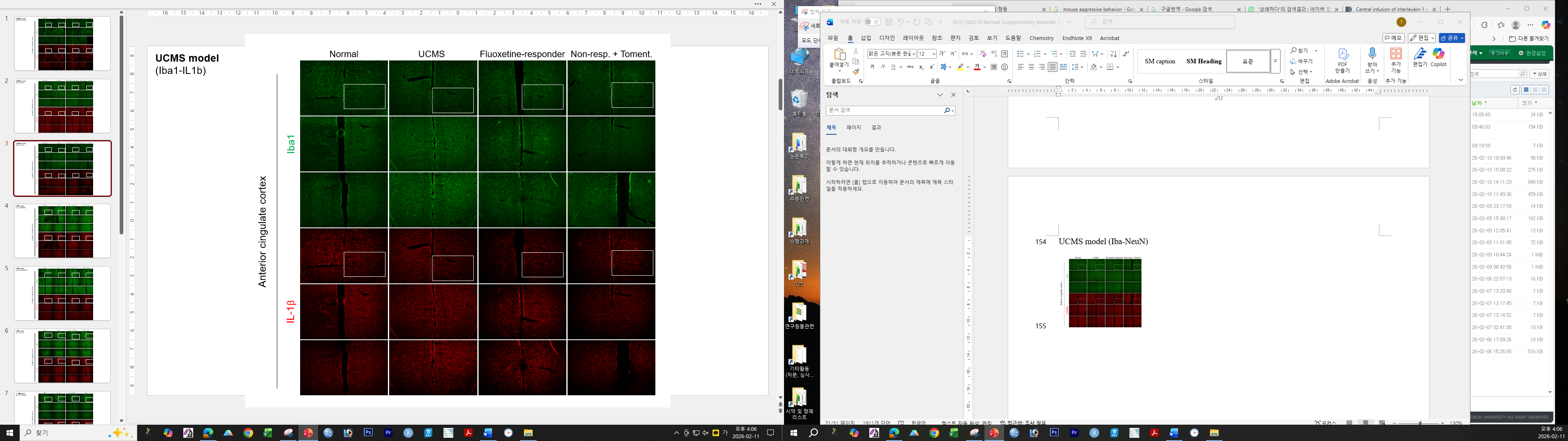


LPS model (Iba-GSDMD)


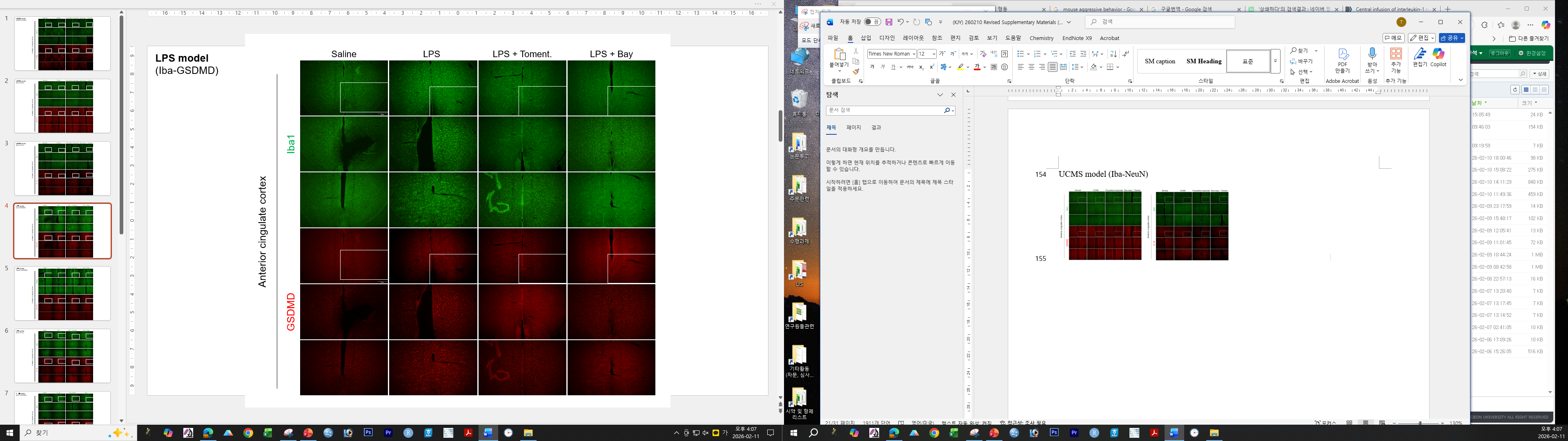


LPS model (NeuN-GSDMD)


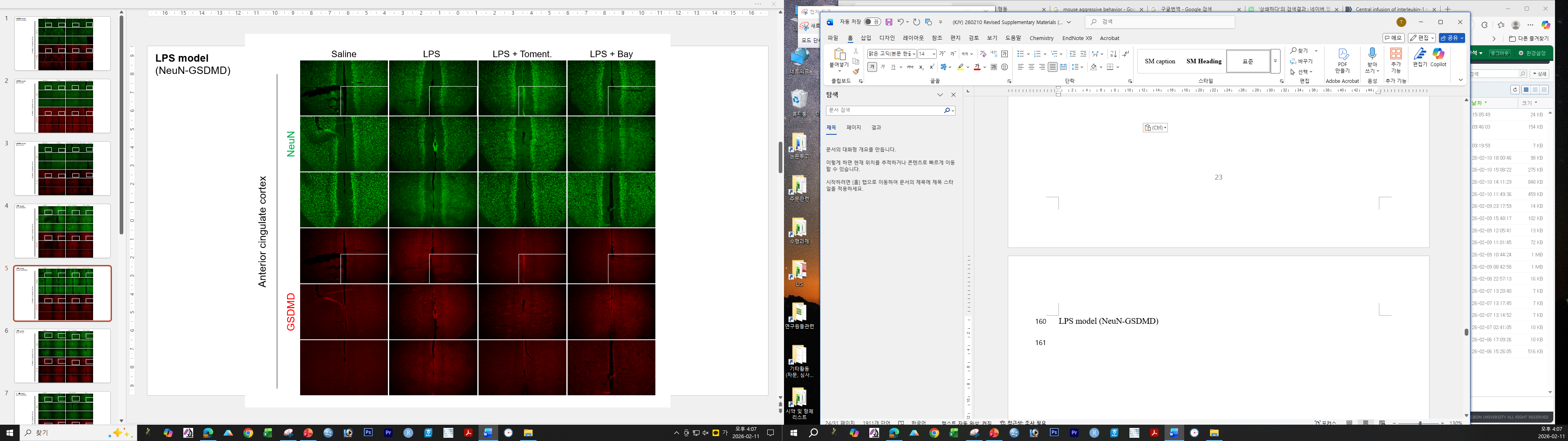


LPS model (Iba1-IL1β)


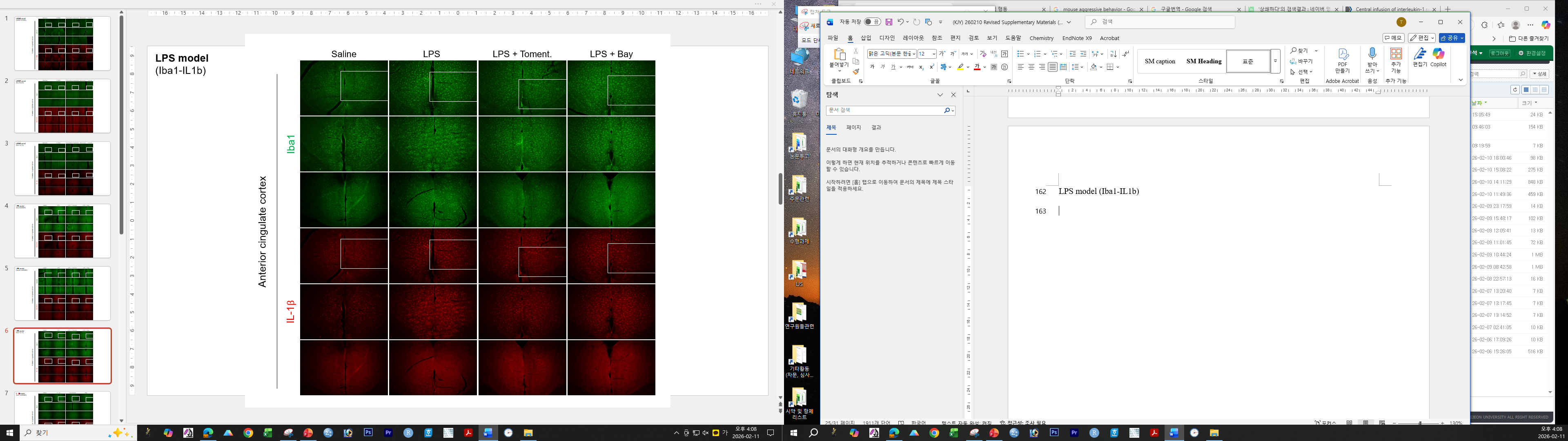


IL-1β model (Iba1-GSDMD)


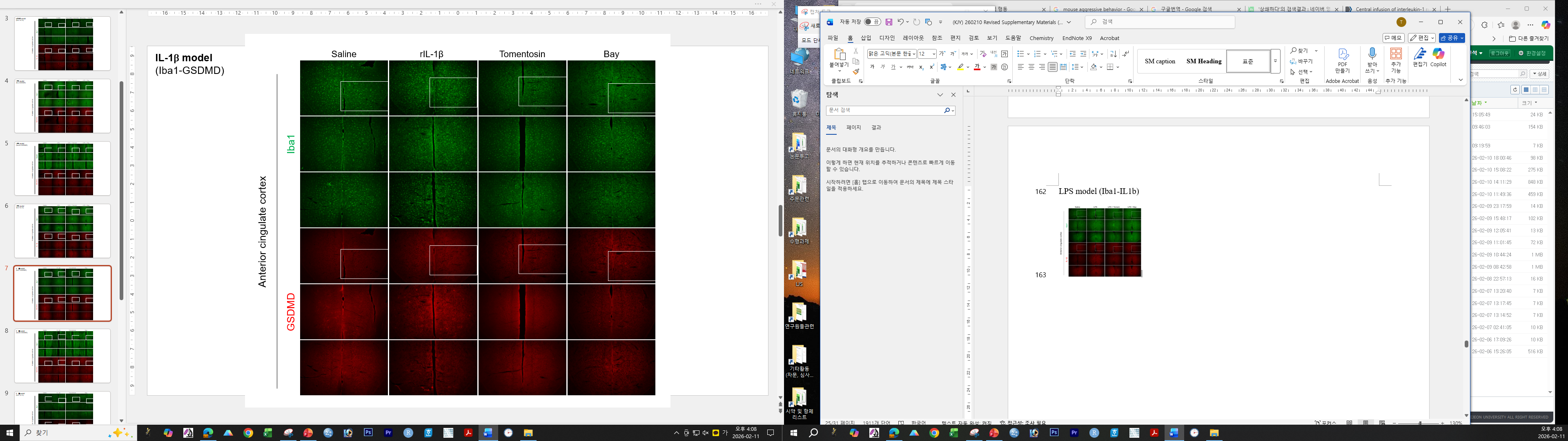


IL-1β model (NeuN-GSDMD)


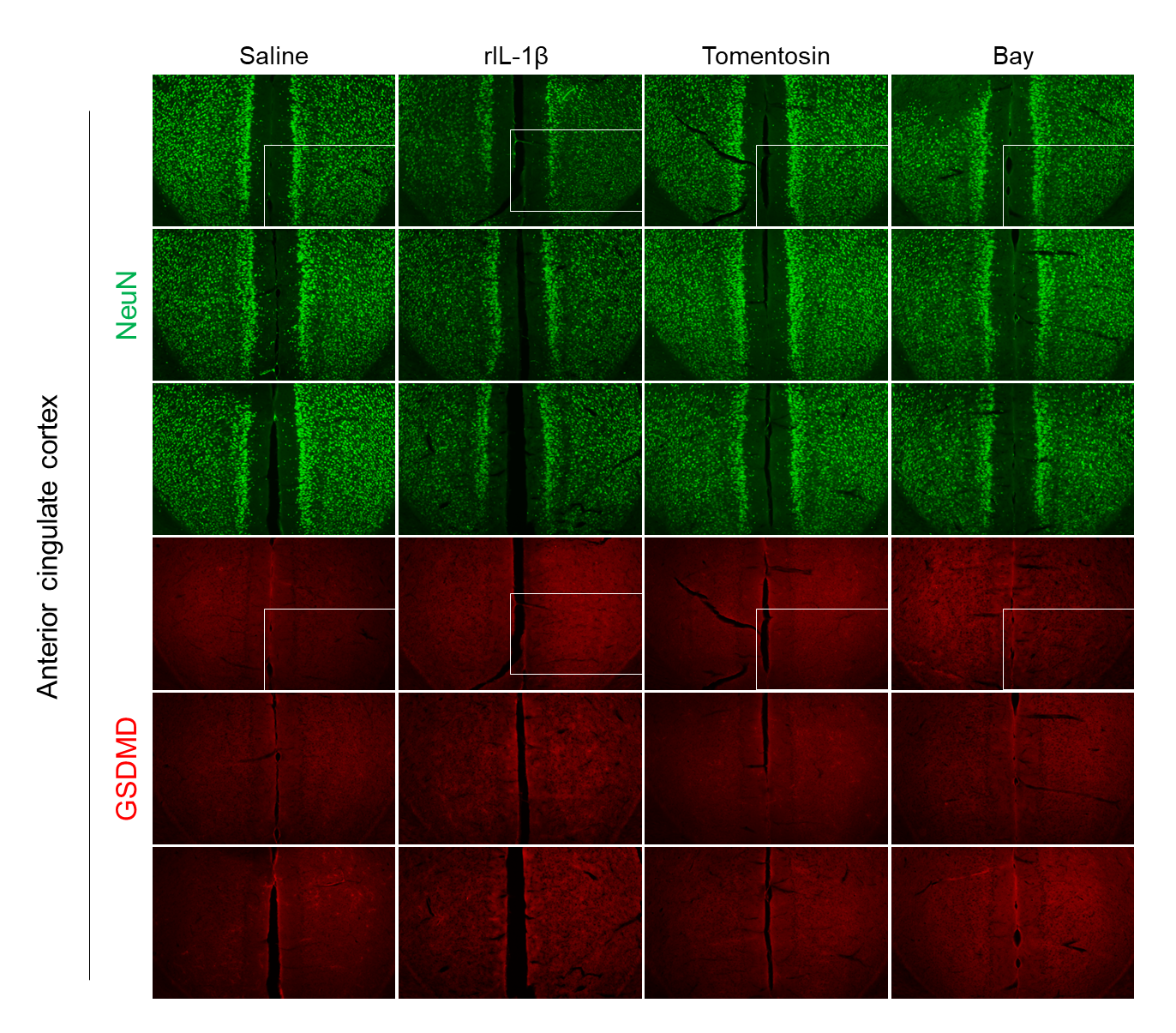


IL-1β model (Iba1-IL1β)


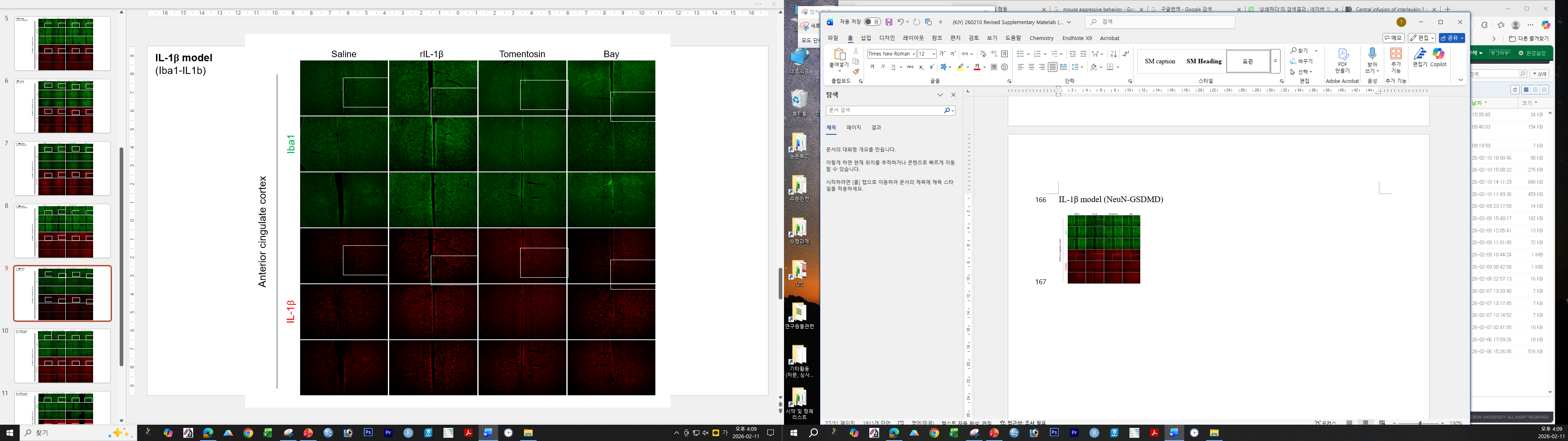


Cas1KO model (Iba1-GSDMD)


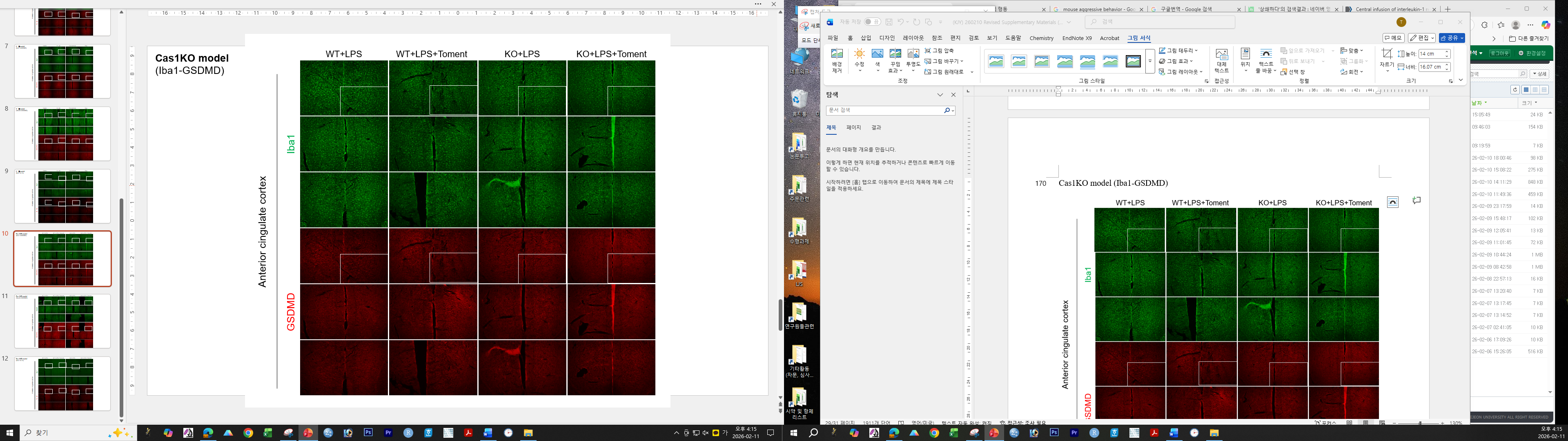


Cas1KO model (NeuN-GSDMD)


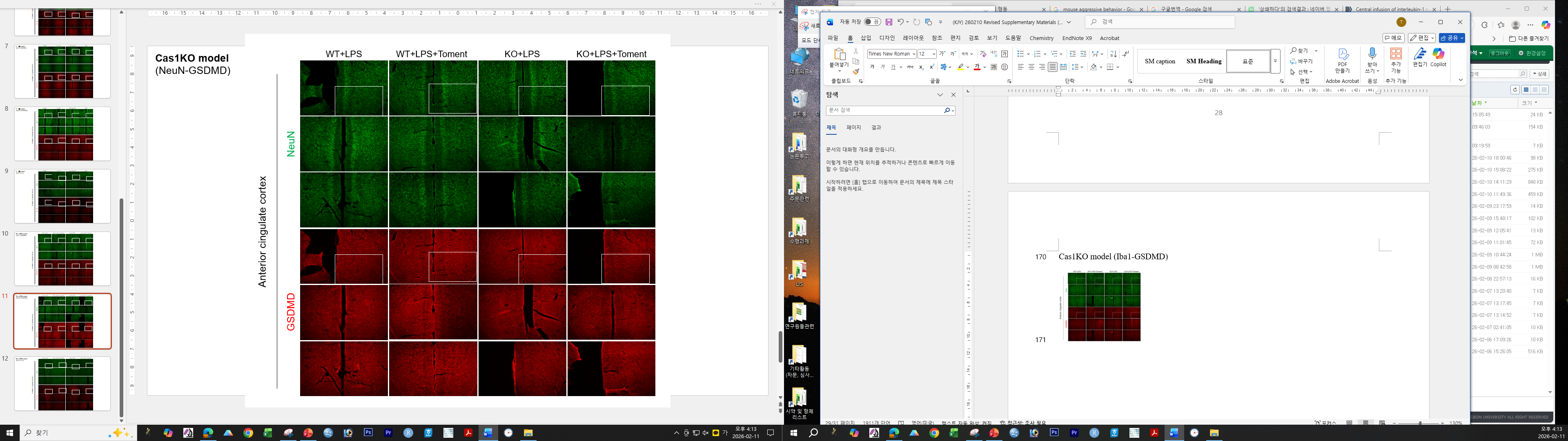


Cas1KO model (Iba1-IL1β)


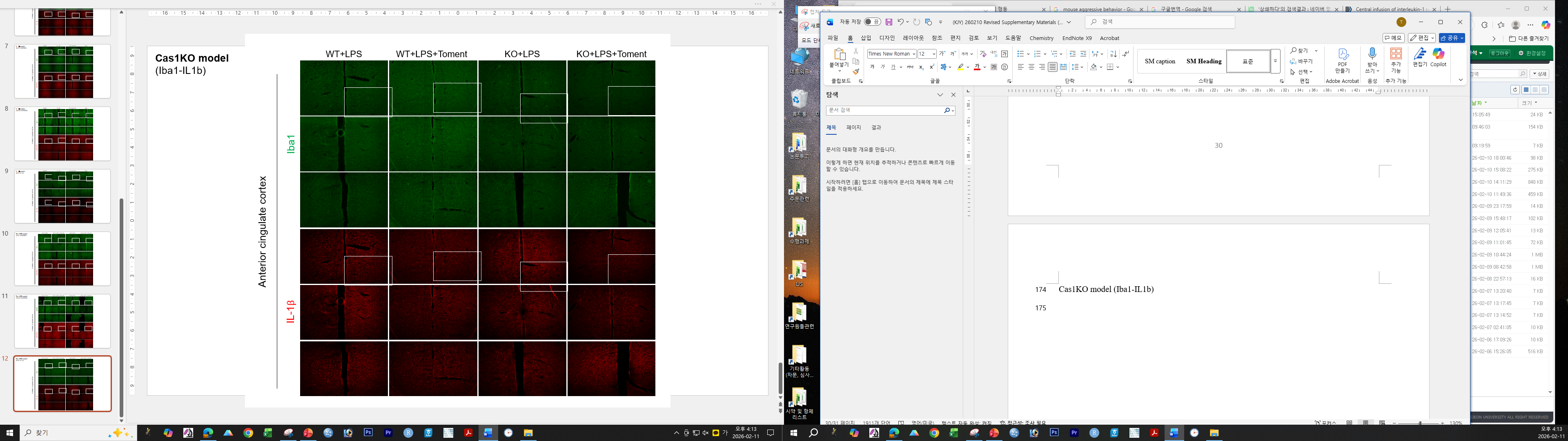

Supplement: Supplementary file 1 — Supplementary Materials [file 41398_2026_4092_MOESM1_ESM.docx]
